# Exploring the potential of gut microbiota metabolites in the treatment of endometriosis through network pharmacology and Mendelian randomization

**DOI:** 10.3389/fmicb.2026.1733323

**Published:** 2026-06-11

**Authors:** Jun Zhou, Tiehu Shan, Yan Li, Guangmei Zhang

**Affiliations:** Department of Gynecology, The First Affiliated Hospital, Harbin Medical University, Harbin, China

**Keywords:** endometriosis, gut microbiota, Mendelian randomization, metabolites, network pharmacology

## Abstract

**Background:**

Studies have shown that dysregulation of the gut microbiota (GM) plays a crucial role in the development of endometriosis (EMs). Study aimed to investigate the potential of protective GM metabolites in treating EM through network pharmacology and Mendelian randomization (MR), opening new avenues for targeted therapeutic strategies.

**Methods:**

All data were sourced from publicly available databases. Biomarkers linked to protective GM metabolites in EMs were identified employing MR study (with GM as the exposure and EMs as the outcome), differential expression analysis, machine learning, and gene expression analyses. Key metabolites associated with these biomarkers were identified through the gutMGene database. A network connecting biomarkers, key metabolites, and key microbes was constructed. Subsequently, drug similarity for the key metabolites was assessed, and molecular docking studies were performed to evaluate potential therapeutic interactions. Finally, reverse transcription quantitative PCR (RT-qPCR) analyses were conducted to further investigate the expression of biomarkers in clinical samples.

**Results:**

The MR study identified 5 key protective microbiota (such as genus.*Bifidobacterium*.id.436) with a causal relationship to EMs (OR < 1, 95% CI ≠ 1, *p* < 0.05). After, AOC3, FABP4, and NEK2 were identified as biomarkers, with AOC3 and FABP4 showing low expression in EMs samples and NEK2 showing higher expression. Based on these biomarkers, 4 key metabolites were identified, and a biomarker-key metabolite-key microbe network was constructed, illustrating relationships such as AOC3-N-acetylputrescine-*Bifidobacterium*. The key metabolites interacting with biomarkers adhere to Lipinski’s rules for pharmacokinetic properties. Molecular docking revealed the binding affinity between biomarkers and key metabolites, with a binding energy of −6.6 kcal/mol for NEK2 and 6-[(4R,5S)-5-methyl-2-oxoimidazolidin-4-yl]hexanoic acid. Finally, RT-qPCR analysis confirmed that the expressions of AOC3 were significantly higher in normal samples compared to case samples (both ectopic and eutopic), while FABP4 expression was significantly higher in normal samples only when compared to eutopic samples.

**Conclusion:**

This study identified AOC3, FABP4, and NEK2 as potential EMs biomarkers associated with gut microbiota-derived metabolites. However, the proposed microbiota-metabolite-biomarker network requires further experimental validation.

## 1 Introduction

Endometriosis (EMs), a prevalent chronic gynecological disorder, is defined by the ectopic proliferation of endometrial tissue—normally restricted to the uterine cavity—in extrauterine regions (Han et al., 2025; Lu et al., 2025). The main symptoms of EMs include pain (such as dysmenorrhea, painful intercourse, etc.), the formation of pelvic masses and nodules, and infertility, which seriously affect women’s quality of life and mental health (Saunders and Horne, 2021; Taylor et al., 2021). According to global statistics, approximately 196 million women are affected by this disease (Zhou et al., 2023a). The pathogenesis of EMs is complex, and its main risk factors include genetic factors (family history), endocrine disorders (such as abnormal estrogen levels), immune dysfunction, imbalance of gut microbiota, and environmental factors (Horne and Missmer, 2022; Vannuccini et al., 2022; Xholli et al., 2023). The treatment methods for EMs mainly include surgery, medication, and assisted reproductive technology (Shi J. et al., 2023). Even in cases where treatment is effective, about 40 to 50% of patients will experience recurrence within five years after treatment. Considering the high recurrence rates and treatment side effects (Peng et al., 2021), in-depth exploration of the molecular mechanisms and new treatment strategies for EMs is in urgent need.

Gut Microbiota (GM) is a complex microbial ecosystem that colonizes the gut (Qiu et al., 2022; Ma et al., 2022). It regulates host immune homeostasis (Fan et al., 2023), nutrient absorption (Shulhai et al., 2024), and intestinal barrier function (Di Tommaso et al., 2021) by metabolizing active products such as short-chain fatty acids (SCFAs) and tryptophan metabolites. In recent years, research has shown a significant correlation between GM disorders and EMs. Studies have shown that the gut microbiota of patients with EMs undergoes significant changes, manifested as decreased diversity, imbalanced microbial composition, and the presence of pathogens. These changes can disrupt immune function, exacerbate inflammatory responses, and lead patients to enter a chronic inflammatory state (Jiang et al., 2021; Xholli et al., 2023). Although existing research has revealed a certain correlation between GM and metabolites and EMs, the mechanism and scope of action of GM metabolites on EMs are still unclear. Therefore, in order to provide a solid theoretical basis for developing more effective EMs treatment strategies, it is urgent to explore this complex relationship in depth.

MR is an analytical approach that employs genetic variation as an instrumental variable to assess the causal relationship between exposure factors and disease outcomes (Birney, 2022; Richmond and Davey Smith, 2022; Dobrijevic et al., 2023).

In recent years, many studies have used MR analysis to reliably evaluate the causal link between GM and EMs (Dang et al., 2024; Su et al., 2024; Yang, 2024). MR analysis can effectively reduce the influence of confounding variables and the possibility of reverse causality by utilizing the random allocation characteristics of genetic variation, providing a reliable basis for further exploring the causal relationship between EMs and GM. Network pharmacology is an emerging branch of pharmacology that, based on the principles of systems biology, reveals the interactions between drugs and the body by constructing and analyzing biological networks (Li et al., 2023; Zhang et al., 2023; Zhao et al., 2023). In recent years, network pharmacology combined with molecular docking technology has been used to study the mechanism of action between GM metabolites and diseases (Oh et al., 2022; Yao et al., 2024). However, the specific mechanism of action between GM metabolites and EMs is still unclear and requires further exploration. This study innovatively combines MR analysis and network pharmacology methods in analyzing the potential association between GM metabolites and EMs, providing new theoretical support and important reference for the development of treatment strategies for EMs.

This study is based on MR data of GM and EMs in the GWAS database, as well as the transcriptome dataset related to EMs in the GEO database. MR analysis, differential expression analysis, machine learning, and gene expression analysis are used in the identification of EMs biomarkers associated with GM metabolites. In addition, we conducted functional exploration and subcellular localization analysis to further explore the potential mechanisms of these biomarkers. Finally, we searched for key metabolites related to biomarkers and constructed a biomarker key metabolite key microbial network. At last, drug like evaluations and molecular docking analyses were conducted on key metabolites. These analyses provide new insights into the treatment of EMs patients.

## 2 Materials and methods

### 2.1 Data collection

This study utilized Mendelian randomization (MR) data for endometriosis (EMs) from the IEU Open GWAS database,^1^ specifically dataset ebi-a-GCST90018839, which included 24,089,752 SNPs from 231,771 European participants (4,511 cases and 227,260 controls; accessed April 25, 2025). Genetic associations for gut microbiota (GM) were obtained from summary statistics provided by the Mibiogen Consortium,^2^ comprising 18,340 individuals across 24 cohorts (85% European ancestry). Using 16S rRNA gene sequencing, taxonomic data were available for 211 microbial groups, spanning 9 phyla, 16 classes, 20 orders, 35 families, and 131 genera.

Transcriptomic data related to endometriosis (EMs) were additionally obtained from the GEO database.^3^ The training dataset GSE7305 (platform: GPL570) included gene expression profiles from 10 EMs cases and 10 control endometrial tissue samples (accessed April 27, 2025). The validation dataset GSE25628 (platform: GPL571) comprised transcriptomic data from 7 ectopic endometrial tissues of EMs patients and 9 control endometrial tissues (accessed April 27, 2025).

### 2.2 Data pre-processing for MR study

A MR analysis was performed using the “TwoSampleMR” package (v0.6.6) (Zhou et al., 2019), with GM serving as the exposure factor and EMs designated as the outcome. The classical MR approach confirmed fulfillment of three fundamental assumptions: (1) the independence assumption, whereby IVs remained uncorrelated with confounding factors; (2) the relevance assumption, wherein IVs exerted direct influence on the exposure variable; and (3) the exclusion—restriction assumption, whereby IVs affected the outcome solely via the exposure variable, devoid of alternative pathways.

Initially, the extract_instruments function was used to retrieve data for the exposure factor and filter the IVs (*p* < 1 × 10^−5^). SNPs in linkage disequilibrium (LD) were then removed (clump = TRUE, R^2^ = 0.001, kb = 10). The number of SNPs was evaluated, and only those with 3 or more SNPs were retained. Subsequently, SNPs associated with the outcome were excluded, while those associated with the exposure factor were kept. Subsequently, effect alleles and effect sizes underwent harmonization via the harmonise_data function. The instrument strength of each genetic variant was rigorously assessed through F-statistics. Following common practice in Mendelian randomization studies, we applied a stringent threshold of *F* > 10 for SNP inclusion. The F-statistic was derived from the following equation:


$$F=\frac{R^{2}\times (n-2)}{1-R^{2}}$$

Where R^2^ indicates the fraction of exposure phenotype variability captured by the IV (i.e., phenotypic variance explained), and *n* stands for the exposure factor GWAS sample size.

### 2.3 MR study, sensitivity analyses, and Steiger test

The MR study was performed by screening IVs and applying five distinct algorithms employing the mr function. These algorithms included IVW (Burgess et al., 2013), MR Egger (Bowden et al., 2015), Weighted Median (Hartwig et al., 2017), Simple Mode (Hemani et al., 2018), and Weighted Mode (Hartwig et al., 2017). Among these, the IVW method was regarded as the most crucial, with the screening criterion for MR set at P_IVW_ < 0.05. At the genus taxonomic rank, we selected putative protective microbial factors meeting all three criteria: OR < 1, 95% CI not spanning 1, and statistically significant PIVW (*p* < 0.05) for downstream investigation. Results were visualized through three plot types: scatter plots (exposure-outcome relationships), forest plots (protective factor efficacy), and funnel plots (effect symmetry analysis).

To ascertain the robustness of the MR findings, multiple sensitivity analyses were conducted. Horizontal pleiotropy was assessed via the MR-Egger function to evaluate potential biases in random estimates stemming from directional pleiotropy, with MR – PRESSO (Lou et al., 2023) further probing this aspect. A *p* > 0.05 suggested no substantial horizontal pleiotropy. Heterogeneity analysis utilized the mr_heterogeneity function (Lu et al., 2022), grounded in Cochran’s Q test, with *p* > 0.05 prompting the fixed IVW approach and p < 0.05 mandating the random IVW method. Leave-one-out (LOO) analysis employed the mr_leaveoneout function (Jin et al., 2022) to test the stability of MR estimates by excluding individual IVs. Lastly, the Steiger test was implemented with the directionality_test function (Leng et al., 2024). To satisfy the Steiger test requirements, two criteria had to be simultaneously fulfilled: first, the analysis needed to demonstrate the correct causal direction (indicated as TRUE), and second, the *p*-value had to show statistical significance below the 0.05 threshold.

Through these comprehensive analyses, key protective microbiota (referred to as key microbiota) were identified for further investigation.

### 2.4 Identification of key microbiota metabolites-target genes and EMs-related target genes

Based on the key microbiota, the gutMGene database (v 2.0) was applied to predict the corresponding metabolites. The SMILES notation for these metabolites was retrieved from PubChem. These SMILES were then input into the Swiss Target Prediction and Similarity Ensemble Approach databases. The final target genes of the key microbiota metabolites (referred to as key microbiota metabolites-target genes) were obtained by integrating the predictions from both databases and removing duplicates.

Additionally, the DisGeNET^4^ and GeneCards^5^ databases were utilized to search for the target genes of EMs, respectively. Notably, the UniProt was used to standardize the target genes (*Homo sapiens*). Lastly, the final set of EMs-related target genes was obtained by integrating and eliminating duplicates from the results of the two databases.

### 2.5 Differential expression analysis

The identification of differentially expressed genes (DEGs) in endometriosis (EMs) versus control samples from the GSE7305 dataset was performed using the “limma” package (version 3.54.1) (Ritchie et al., 2015), applying stringent thresholds of absolute log₂ fold-change > 1.5 and adjusted *p*-value < 0.05 for significance. Subsequently, the identified DEGs were illustrated through volcano plots and heatmaps, constructed via the “ggplot2” package (v3.4.1) (Gustavsson et al., 2022) and the “ComplexHeatmap”package (v2.14.0) (Gu, 2022) packages, respectively.

### 2.6 Identification and function analyses of candidate genes

Candidate genes for downstream analysis were determined by intersecting three gene sets: DEGs, microbiota metabolite-target genes, and EMs-associated genes, using the “ggvenn” package (v0.1.9) (Zhou et al., 2025). These overlapping genes were subsequently subjected to functional enrichment analysis (GO and KEGG pathways) through the “clusterProfiler” tool (v4.2.2) (Yu et al., 2012), with statistical significance set at *p* < 0.05.

To further explore protein–protein interactions (PPIs) among candidate genes, this study constructed a PPI network based on the STRING database.^6^ In order to comprehensively capture potential protein–protein interactions, the PPI network was analyzed with a confidence score of >0.15, which was set as the screening threshold by referring to the official minimum confidence threshold of STRING and relevant published studie (Szklarczyk et al., 2023). The resultant PPI network was visualized distinctly using Cytoscape (v 3.7.2) (Shannon et al., 2003).

### 2.7 Determination of biomarkers and construction of a biomarker-key metabolite-key microbe network

The candidate gene set was analyzed using two distinct machine learning approaches—the Boruta feature selection algorithm and random forest (RF) classification-to identify robust biomarker candidates. Specifically, the “Boruta” package (v 8.0.0) (Maurya et al., 2023) was applied to perform the Boruta algorithm, and genes were selected based on their importance (*p* = 0.01, mcAdj = T, maxRuns = 30). For RF, the “randomForest” package (v 3.2.3) (Wang et al., 2023) was utilized. The overlapping genes selected by both Boruta and random forest algorithms were determined through visualization with the “ggvenn” package (version 0.1.9), enabling systematic identification of potential biomarkers. We systematically analyzed expression profiles of potential biomarkers in the GSE7305 and GSE25628 datasets through nonparametric Wilcoxon testing (*p* < 0.05). Only genes meeting dual criteria—statistically significant differential expression in EMs versus controls (*p* < 0.05) and consistent expression patterns in both independent datasets—were classified as validated biomarkers.

Following the identification of biomarkers, key metabolites targeting these biomarkers were retrieved from the gutMGene database, and a biomarker-key metabolite-key microbe network was constructed and visualized employing Cytoscape (v 3.7.2).

### 2.8 Function exploration and subcellular localization of biomarkers

To uncover potential interactions and functional links between the biomarkers and other genes, the GeneMANIA database^7^ was leveraged. Additionally, to determine the subcellular localization of the biomarkers, their FASTA sequences were retrieved from the NCBI database. Afterward, mRNALocater^8^ was used to forecast the subcellular localization of these biomarkers.

### 2.9 Pharmacological evaluation and molecular docking

In this study, SwissADME^9^ and ADMETlab^10^ were employed to conduct pharmacological assessment of key metabolites targeting the biomarkers. Their pharmacological properties were assessed based on Lipinski’s five rules: (1) molecular weight (MW) < 500; (2) lipid-water partition coefficient (LogP) < 5; (3) ≤ 9 hydrogen bond acceptors (HBA); (4) ≤ 4 hydrogen bond donors (HBD); and (5) topological polar surface area (TPSA) < 140. To investigate potential interactions, molecular docking was performed between key metabolites (ligands) and identified biomarkers (receptors). Metabolite structures were retrieved from PubChem, while biomarker protein structures were sourced from the PDB database.^11^ Molecular docking analyses were performed using the CB-Dock platform,^12^ with binding energy calculated. A binding energy of < −5 kcal/mol typically suggests strong binding affinity between key metabolites and biomarkers, implying potential for effective molecular interaction.

### 2.10 Clinical sample collection

Patients with ovarian EM who underwent surgery at the First Affiliated Hospital of Harbin Medical University from July 2021 to June 2024 were enrolled. The eutopic endometrium tissues (EU) and ovarian endometriosis tissues (EC) were collected in a total of 5 cases. Moreover, 5 cases of normal control endometrium tissues (NM) diagnosed as cervical lesions or uterine myoma were collected. Specimens in all 3 groups were excluded hormone treatment for nearly six months. All tissues were verified by histopathologists.

### 2.11 Reverse transcription and RT-qPCR

Total RNA from patients was extracted with TRIzol reagent (Ambion, United States) and converted to complementary DNA using a PrimeScript^™^ RT reagent Kit with gDNA Eraser (Takara, Japan). RT-qPCR was performed with SYBR Green qPCR SuperMix (Vazyme, China). The settings were as follows: 40 cycles of 95 °C 5 min, 95 °C 15 s, 60 °C 32 s. All relative mRNA expression levels were analyzed using the 2 − ∆∆Ct method. The primers used are listed in Supplementary Table S1.

### 2.12 Statistical analysis

R software (v 4.2.0) was employed for statistical analyses. Between-group differences were assessed via the Wilcoxon test (*p* < 0.05). All experiments were repeated three times, and statistical work was carried out using GraphPad Prism 9.0 and SPSS software. The results are presented as the mean ± standard deviation. A comparison of all experimental results between two groups was performed using Student’s t-test, or one-way ANOVA among three or more groups. The difference was considered significant at *p* < 0.05 (ns, *p* ≥ 0.05; *, *p* < 0.05; **, *p* < 0.01).

## 3 Results

### 3.1 Revelation of robust causal relationships between 5 key microbiota and EMs

**Figure 1:**
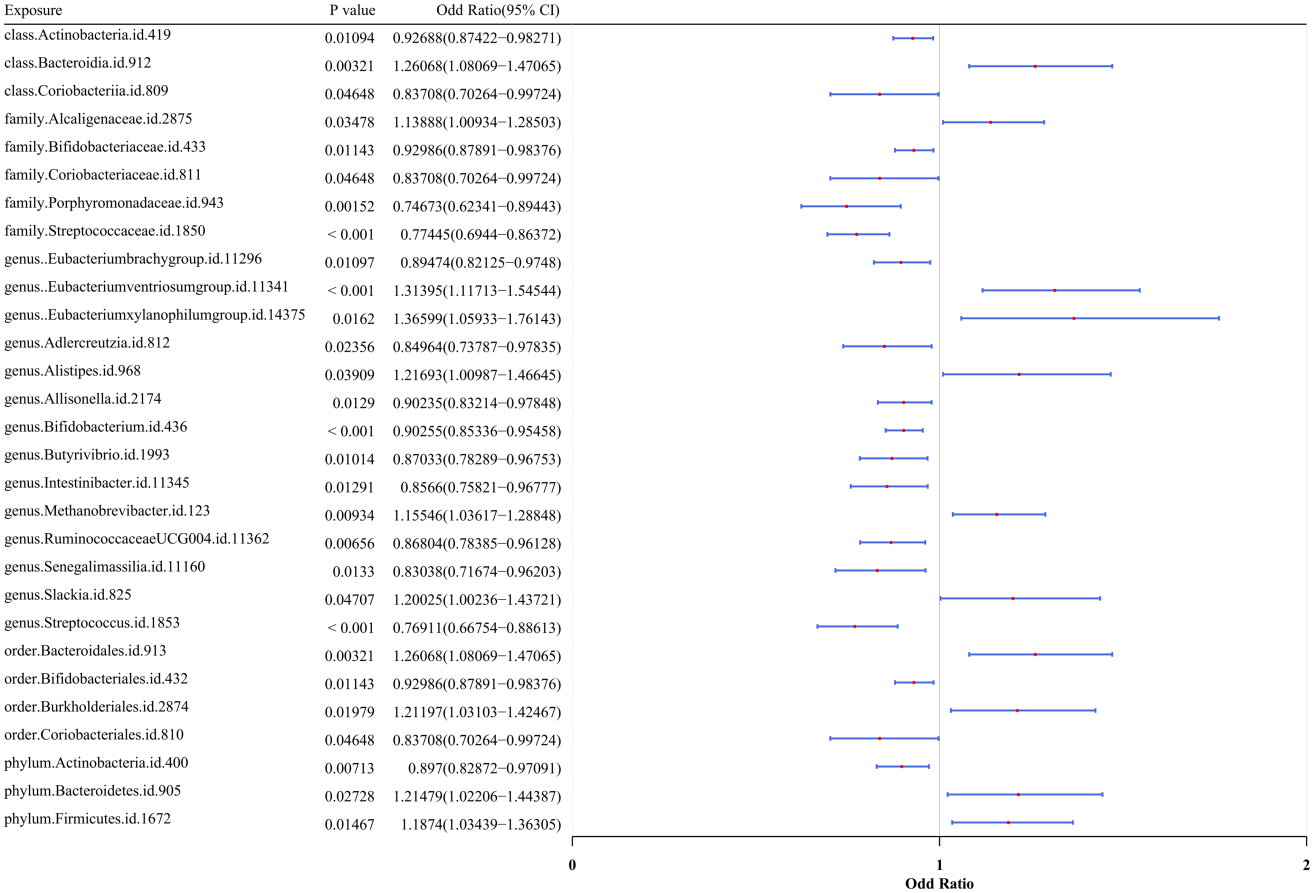
Forest plot illustrating the causal associations between microbiota and EMs identified by MR analysis.

In this study, through MR analysis, sensitivity analysis, and the Steiger test, five key microbiota with robust causal relationships to EMs were identified. Initially, through MR analyses, significant causal relationships were identified between 14 microbiota (at the genus level) and EMs (*p* < 0.05) (Figure 1). Notably, 9 microbiota, such as genus.*Allisonella*.id.2174 (OR = 0.9023, 95% CI = 0.8321–0.9785, *p* = 0.0129) and genus.*Bifidobacterium*.id.436 (OR = 0.9026, 95% CI = 0.8534–0.9546, *p* = 0.0003), exhibited protective effects (Table 1).

**Table 1:** The 9 protective microbiota associated with EMs.

ID exposure	ID outcome	nsnp	*P*	OR	OR_lci95	OR_uci95
genus.*Eubacteriumbrachygroup*.id.11296	Endometriosis	19	0.0110	0.8947	0.8213	0.9748
genus.*Adlercreutzia*.id.812	Endometriosis	14	0.0236	0.8496	0.7379	0.9783
genus.*Allisonella*.id.2174	Endometriosis	19	0.0129	0.9023	0.8321	0.9785
genus.*Bifidobacterium*.id.436	Endometriosis	88	0.0003	0.9026	0.8534	0.9546
genus.*Butyrivibrio*.id.1993	Endometriosis	31	0.0101	0.8703	0.7829	0.9675
genus.*Intestinibacter*.id.11345	Endometriosis	25	0.0129	0.8566	0.7582	0.9678
genus.*RuminococcaceaeUCG004*.id.11362	Endometriosis	30	0.0066	0.8680	0.7838	0.9613
genus.*Senegalimassilia*.id.11160	Endometriosis	10	0.0133	0.8304	0.7167	0.9620
genus.*Streptococcus*.id.1853	Endometriosis	48	0.0003	0.7691	0.6675	0.8861

The subsequent results chart focused exclusively on the five key microbiota identified through comprehensive analysis. Scatter plots of these protective microbiota showed negative regression slopes (Supplementary Figure S1), and forest plot analysis employing the IVW method revealed consistently negative effect sizes (MR effect sizes < 0) (Supplementary Figure S2). Additionally, funnel plots displayed a symmetric distribution of IVs around the IVW line, supporting the adherence to Mendel’s second law (Supplementary Figure S3). The horizontal pleiotropy analysis revealed nonsignificant results (*p* > 0.05) for all six protective microbiota, indicating absence of detectable horizontal pleiotropic effects (Supplementary Table S2). However, for 3 protective factors (genus.*Eubacteriumbrachygroup*.id.11296, genus.*Butyrivibrio*.id.1993, and genus.*Streptococcus*.id.1853), *p* values were less than 0.05, prompting their exclusion from further analysis. The remaining 6 protective microbiota showed no evidence of heterogeneity (*p* > 0.05), and thus were analyzed employing the fixed IVW method (Supplementary Table S3). Moreover, LOO analysis demonstrated that the exclusion of genus.*Adlercreutzia*.id.812 for each SNP caused a significant change in the effect of the remaining SNP on the outcome variable, suggesting that the MR results were unstable and unreliable (Supplementary Figure S4). Therefore, genus.*Adlercreutzia*.id.812 was excluded, and the remaining 5 protective microbiota were subjected to further analysis. The Steiger test of directionality provided robust evidence supporting a causal relationship between these five protective microbial factors and EMs, with both correct directional orientation (TRUE) and extreme statistical significance (*p* < 0.0001) (Supplementary Table S4). In summary, comprehensive analyses identified 5 key microbiota (genus.*Allisonella*.id.2174, genus.*Bifidobacterium*.id.436, genus.*Intestinibacter*.id.11345, genus.*RuminococcaceaeUCG004*.id.11362, and genus.*Senegalimassilia*.id.11160) that exhibited strong causal relationships with EMs, highlighting their potential as protective factors in the context of EMs. In addition, although the obvious pleiotropy interference has been effectively controlled and excluded by multiple sensitivity analyses, and the results of all four types of tests showed *p* > 0.05, the possibility of residual pleiotropy cannot be completely ruled out. MR analysis relies on the core assumption that instrumental variables affect outcomes only through exposure factors, which is difficult to validate absolutely in complex biological systems.

### 3.2 Determination and function exploration of the 12 candidate genes

Based on the 5 key microbiota, only genus. *Bifidobacterium*.id.436 was found to predict the corresponding metabolites in the gutMGene database. Consequently, metabolites associated with *Bifidobacterium* were identified by searching the gutMGene database. SMILES data, obtained from the PubChem database, were then input into the Swiss Target Prediction and Similarity Ensemble Approach databases to identify 461 key microbiota metabolites-target genes (Supplementary Table S5). Furthermore, 3,394 EMs-related target genes were identified by integrating results from the DisGeNET and GeneCards databases (Supplementary Table S5).

**Figure 2:**
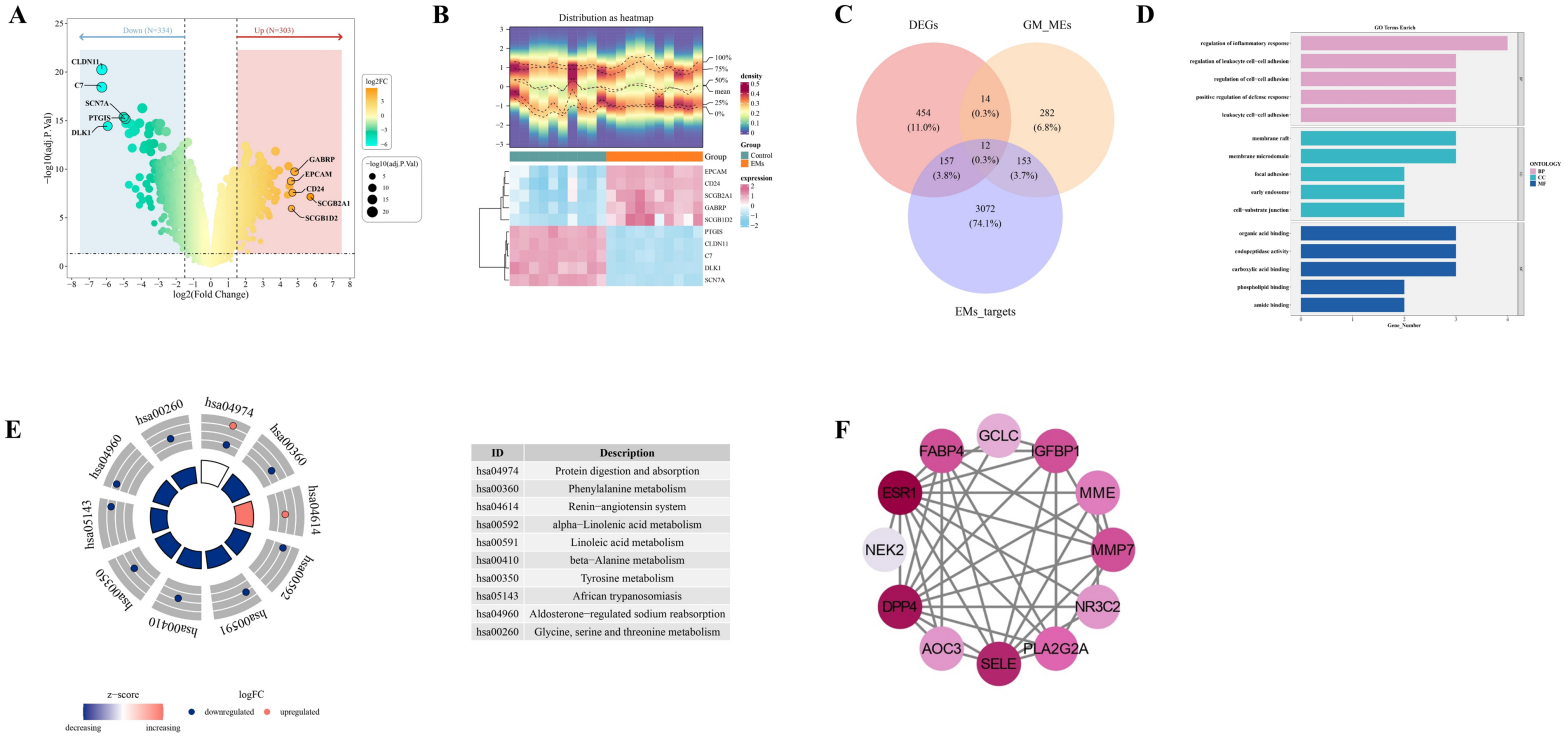
**(A)** Differential expression analysis of genes between EMs and control samples (volcano plot). **(B)** Heatmap and density distribution of DEGs between EMs and control samples. **(C)** Venn diagram illustrating the intersection of DEGs, GM_MEs, and EMs-related target genes to screen candidate genes. **(D)** GO enrichment analysis of candidate genes, showing top 5 terms in BP, CC, and MF categories. **(E)** KEGG pathway enrichment analysis of candidate genes, displaying top 10 pathways ranked by *p*-value (circular plot and table). **(F)** Protein–protein interaction (PPI) network of candidate genes, illustrating protein-level interactions.

Additionally, 637 DEGs were identified between EMs and control samples, including 303 up- and 334 down-regulated genes (Figures 2A,B). The 12 candidate genes were then determined by overlapping the 461 key microbiota metabolites-target genes, 3,394 EMs-related target genes, and 637 DEGs (Figure 2C). The 12 candidate genes were notably associated with 332 GO terms, including 267 BPs, 11 CCs, and 54 MFs (Supplementary Table S6). The top five terms in each category (BP, CC, MF), ranked by count from highest to lowest, included “regulation of inflammatory response” (BP), “membrane microdomain” (CC), and “endopeptidase activity” (MF), etc. (Figure 2D). KEGG pathway analysis demonstrated significant enrichment (*p* < 0.05) of the 12 candidate genes across 13 metabolic and signaling pathways (Supplementary Table S6). The top 10 pathways, ranked by *p* value from lowest to highest, included “protein digestion and absorption,” “phenylalanine metabolism,” and “renin-angiotensin system,” among others (Figure 2E). A PPI network further illustrated the interactions between these candidate genes at the protein level (Figure 2F). ESR1 demonstrated robust molecular interactions with multiple genes, including MMP7 and SELE.

### 3.3 Identification of AOC3, FABP4, NEK2 as biomarkers for EMs

**Figure 3:**
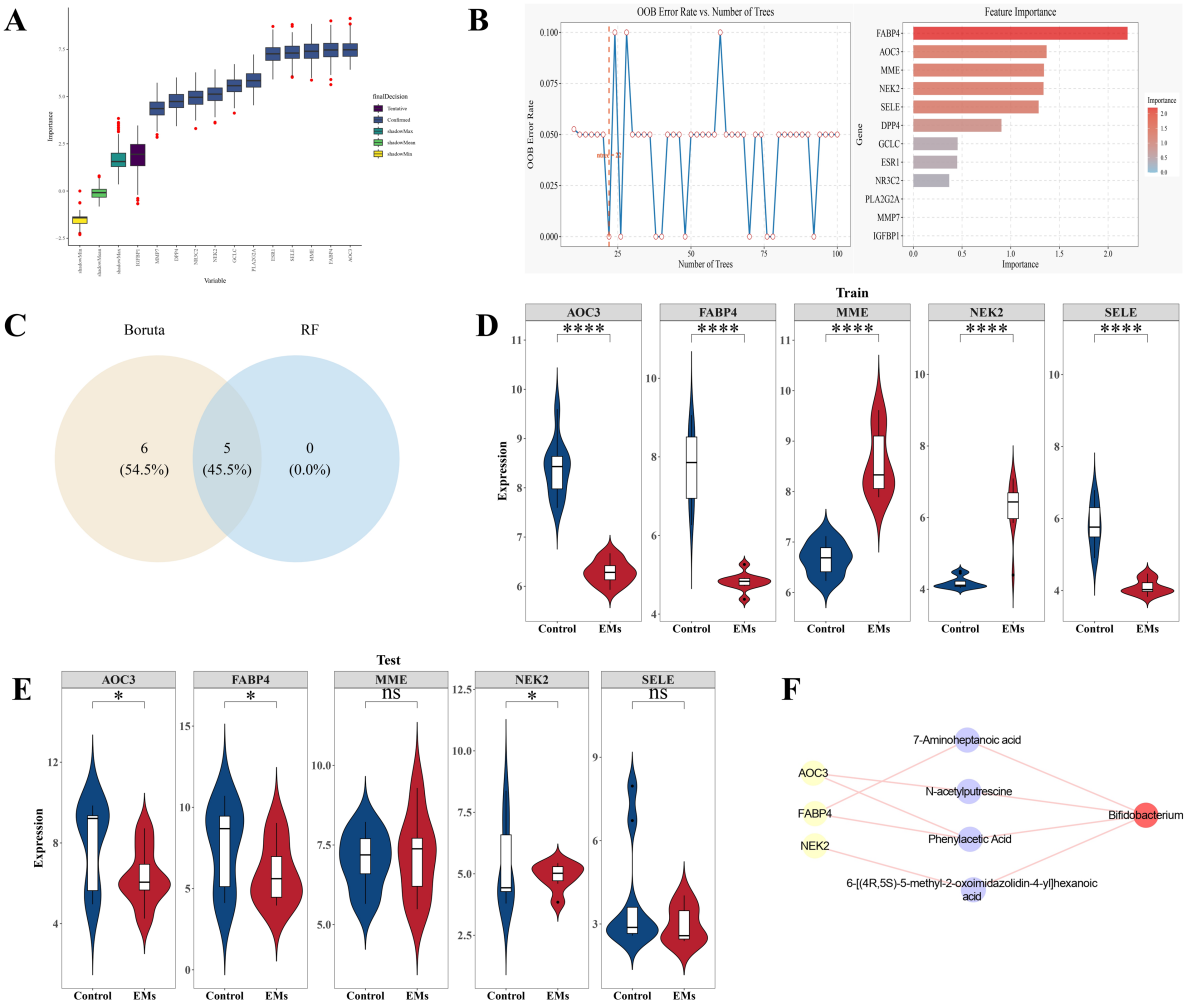
Illustrates the stepwise identification of biomarkers and their microbial-metabolic interactions in EMs. **(A)** The Boruta algorithm filtered 11 genes from 12 candidates. **(B)** RF analysis (ntree = 22) prioritized the top 5 most important genes, visualized via OOB error curves and feature importance plots. **(C)** A Venn diagram depicting the intersection of 11 Boruta-identified genes and 5 RF-prioritized genes defined 5 candidate biomarkers. **(D,E)** Violin plots showed differential expression of AOC3, FABP4, NEK2, and other genes in EMs vs. control samples across GSE7305 **(D)** and GSE25628 **(E)** datasets (*p* < 0.05). **(F)** A biomarker-key metabolite-key microbe interaction network revealed relationships, e.g., AOC3-N-acetylputrescine-Bifidobacterium, FABP4-7-aminoheptanoic acid-Bifidobacterium, and NEK2-6-[(4R,5S)-5-methyl-2-oxoimidazolidin-4-yl] hexanoic acid-Bifidobacterium.

Based on the 12 candidate genes, 11 genes were determined by Boruta (Figure 3A), and the top 5 most important genes were determined by RF analysis (ntree = 22) (Figure 3B). Subsequently, 5 candidate biomarkers were determined by overlapping the 11 genes from Boruta with the 5 genes from RF (Figure 3C). AOC3 and FABP4 exhibited significantly lower expression in EMs samples, while NEK2 showed significantly higher expression in EMs samples across both the GSE7305 and GSE25628 datasets (*p* < 0.05) (Figures 3D,E). These findings highlighted AOC3, FABP4, and NEK2 as biomarkers in the pathogenesis of EMs, suggesting their potential as therapeutic targets for disease management. Additionally, 4 types of key metabolites associated with these biomarkers were identified. A predicted biomarker-key metabolite-key microbe network was constructed based on database-derived associations, revealing putative relationships such as AOC3-N-acetylputrescine-*Bifidobacterium*, AOC3-phenylacetic acid-*Bifidobacterium*, FABP4-7-aminoheptanoic acid-*Bifidobacterium*, FABP4-7-phenylacetic acid-*Bifidobacterium*, and NEK2-6-[(4R,5S)-5-methyl-2-oxoimidazolidin-4-yl] hexanoic acid-*Bifidobacterium* (Figure 3F). These relationships require further experimental confirmation.

### 3.4 Comprehensive analyses of biomarkers

**Figure 4:**
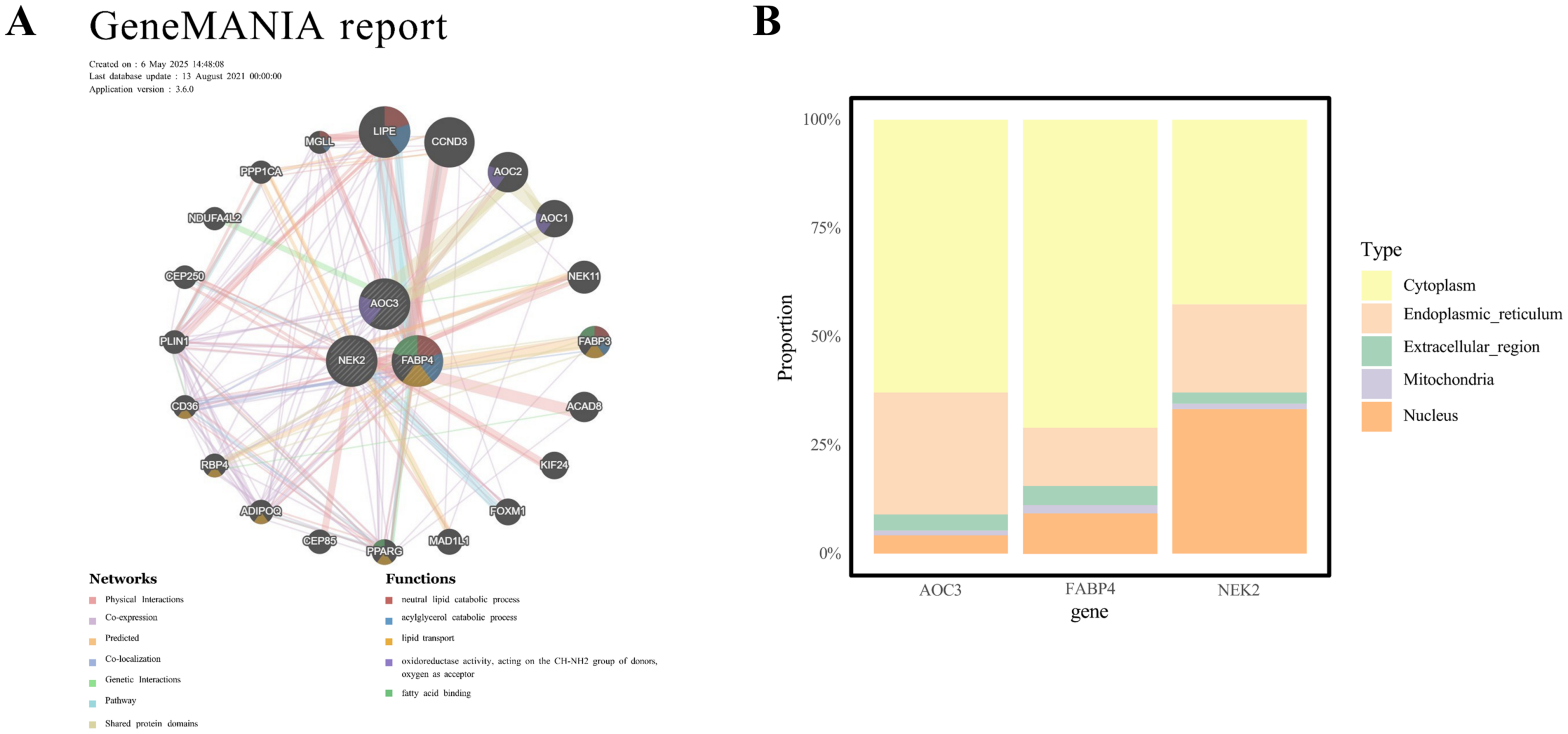
**(A)** GeneMANIA-based functional interaction network of biomarkers, identifying 20 associated genes (e.g., AOC3–AOC1, FABP4–FABP3, NEK2–CEP85) involved in processes like neutral lipid catabolism, acylglycerol catabolism, and lipid transport. **(B)** Subcellular localization analysis showing the predominant cytoplasmic distribution of AOC3, FABP4, and NEK2.

Using the GeneMANIA platform, 20 genes functionally correlated with the biomarkers were uncovered. For instance, AOC3 was connected with AOC1, FABP4 was associated with FABP3, and NEK2 was linked to CEP85. These genetic interactions participated in key biological processes, including the “neutral lipid catabolic process,” “acylglycerol catabolic process,” and “lipid transport” (Figure 4A). Analysis of subcellular localization showed that AOC3, FABP4, and NEK2 were mainly localized in the cytoplasm (Figure 4B). These findings offered deeper insights into the molecular mechanisms underpinning EMs and implied that these genes could exert pivotal functions in lipid metabolism and transport, potentially impacting disease progression.

### 3.5 Exploration the potential treatment mechanisms of EMs

**Figure 5:**
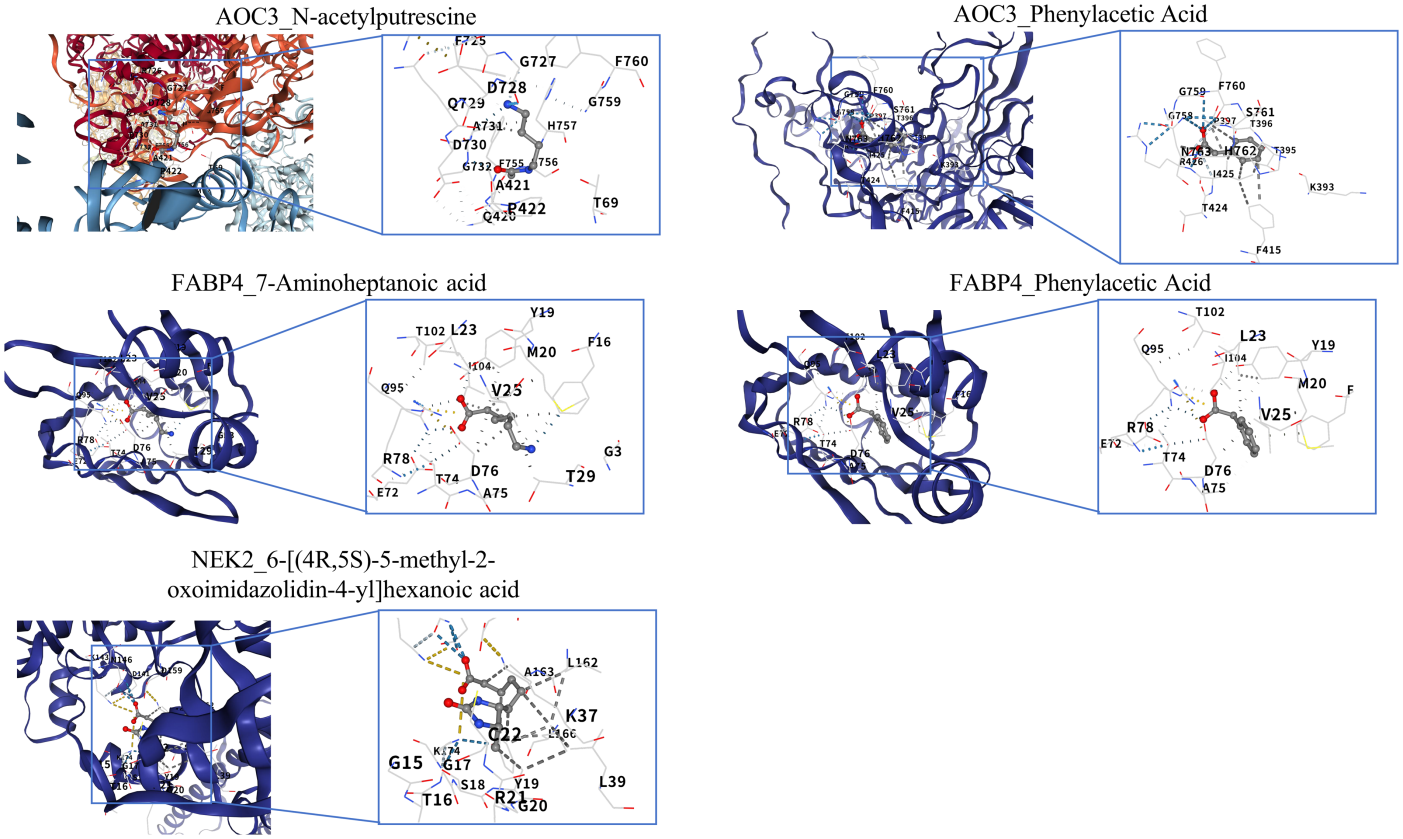
Molecular docking analysis of biomarkers and their key metabolites.

The key metabolites interacting with biomarkers adhere to Lipinski’s rules for pharmacokinetic properties, indicating that they possess the essential physical, chemical, and structural characteristics required to be considered as potential drug candidates. They meet fundamental criteria such as oral bioavailability, metabolic stability, and safety (Table 2). To further investigate these interactions, molecular docking analysis was performed on biomarkers and their corresponding key metabolites (Figure 5). Molecular docking analysis demonstrated favorable binding between AOC3 and phenylacetic acid (binding energy: −6.0 kcal/mol), with key interactions at residues H762 and F760. Moderate binding was observed for AOC3-N-acetylputrescine (−4.9 kcal/mol), while FABP4 exhibited promising affinity for phenylacetic acid (−5.4 kcal/mol), suggesting potential pharmacological relevance. The binding energy between FABP4 and 7-aminoheptanoic acid was relatively weak at −4.3 kcal/mol. Notably, NEK2 exhibited the most favorable binding energy of −6.6 kcal/mol with 6-[(4R,5S)-5-methyl-2-oxoimidazolidin-4-yl] hexanoic acid. This interaction was further characterized by key contacts at residues C22 and K37, among others. Overall, molecular docking suggested potential interactions between selected metabolites and host biomarkers, although these findings still require further experimental validation.

**Table 2:** Drug similarity assessment of key metabolites.

Key metabolites	MW (g/mol)	LogP	HBA	HBD	TPSA
6-[(4R,5S)-5-methyl-2-oxoimidazolidin-4-yl]hexanoic acid	214.26	0.82	3	3	78.43
7-Aminoheptanoic acid	145.2	0.14	3	2	63.32
N-acetylputrescine	130.19	0.11	2	2	55.12
Phenylacetic acid	136.15	1.43	2	2	37.3

### 3.6 The experimental validation of AOC3, FABP4, NEK2 as biomarkers for EMs

**Figure 6:**
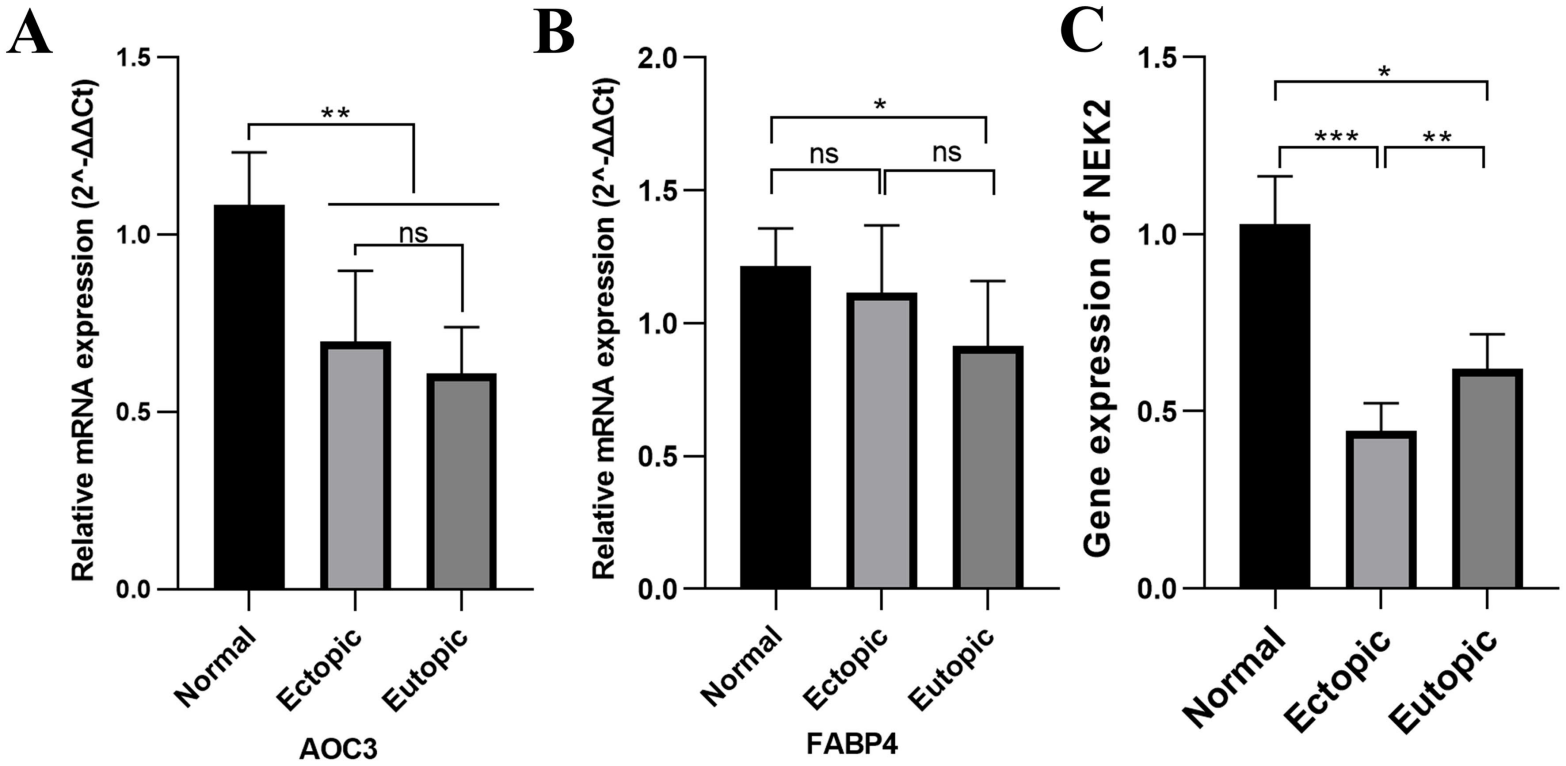
RT-qPCR analysis of key biomarkers AOC3 **(A)**, FABP4 **(B)**, and NEK2 **(C)** in normal, ectopic, and eutopic endometrial samples. Significant differences between groups are indicated. Data represent mean ± SD. *p < 0.05, **p < 0.01, ***p < 0.001, ns = not significant.

To verify the key biomarkers, AOC3, FABP4, NEK2, we employed a quantitative assay to detect AOC3, FABP4, NEK2 alteration experimentally in EM. We observed that the quantity of AOC3 increased significantly in normal endometrium species, while it decreased both in both ectopic and eutopic endometrium species, and decreased the least in the eutopic group (Figure 6A). The RT-qPCR experiment also showed the significant difference in FABP4, NEK2. Although FABP4 showed an altered expression between normal and endometiosis species, there was only significant difference between the normal and eutopic groups (Figure 6B). However, the experiments revealed RT-qPCR experiment showed that the NEK2 expression decreased significantly in both ectopic and eutopic endometrium species (Figure 6C), which is opposite to our exploration above. These findings provide preliminary support for the dysregulated expression of AOC3 and FABP4 in EMs, whereas the inconsistent expression pattern of NEK2 indicates that further validation is required.

## 4 Discussion

EMs impacts 5–10% of women of reproductive age globally, with existing drug therapies showing variable efficacy, highlighting the need for novel treatment strategies (Koninckx et al., 2021); Studies have demonstrated that gut microbiota and its metabolites may be involved in the disease’s pathological mechanisms via immune cell regulation (Qiu et al., 2022). Exploring the relationship between the two and intervening in gut microbiota for treatment is expected to bring breakthroughs in diagnosis and treatment. This study identified 5 protective gut microbiota microorganisms that have a significant causal relationship with EMs through MR analysis. In addition, three biomarkers (AOC3, FABP4, NEK2) were identified. Based on these biomarkers, we predicted four key metabolites associated with them (6- [(4R, 5S)-5-methyl-2-oxoimidazolidin-4-yl] hexanoic acid, 7-Aminoheptanoic acid, N-acetylputrescine, Phenyletic Acid). A key biomarker key metabolite key microbial network was further constructed. Finally, through drug like evaluation and molecular docking analysis, we explored the potential of these metabolites as candidate drug molecules.

Research has found that bifidobacteria exhibit significant protective effects (OR<1, *p* < 0.05), which are consistent with Yang’s (2024) Mendelian randomization (MR) study and support the possible protective association between Bifidobacterium and EMs (Yang, 2024). However, it should be clear that the network pharmacology approach used in this study is inherently highly dependent on the completeness and comprehensiveness of annotations in existing databases. As mentioned in the results section, the current gutMGene database has obvious lack of metabolite annotation information for most microbial taxa except Bifidobacteria, resulting in only effective prediction of bifidobacteria metabolites in this study. This critical limitation can lead to the missing of potential metabolites of other key protective microorganisms, and the predicted molecular interactions will favor well-studied and well-annotated gene and signaling pathways. This high dependence on the database and the risk of false negative or false positive results may affect the comprehensiveness and accuracy of the network analysis in this study to a certain extent.

Bifidobacteria, as a dominant symbiotic bacteria in the intestine, produces metabolites (such as indole derivatives) that have important physiological functions (Rivière et al., 2016; Qian et al., 2024). These metabolites can promote the secretion of IL-22 by activating the aromatic hydrocarbon receptor (AhR) pathway, and IL-22 can effectively enhance mucosal barrier function (Monteleone et al., 2011; Zhang et al., 2024). This mechanism may be one of the important pathways through which bifidobacteria exerts their inhibitory effect. At the same time, clinical studies have provided strong evidence that the abundance of bifidobacteria in the gut of patients with EMs is significantly negatively correlated with disease staging (Iavarone et al., 2023; Sessa et al., 2024; Datkhayeva et al., 2025). This finding suggests that bifidobacteria may inhibit the formation of ectopic lesions by regulating local immune homeostasis, providing richer clinical evidence for a deeper understanding of the role of bifidobacteria in EMs. In addition, Wei et al. (2023)’s report showed that in their species difference analysis of EM versus control samlpes, nine of 10 species showing proportional increases were associated with EMs samples, with only Senegalimassilia being proportionally higher in controls. This is consistent with the results of our study in which Senegalimassilia was found to be a protective gut flora, thus indicating the plausibility of our study. What’s more, we performed GO functional annotation on the core target genes and found that in terms of biological processes, the core target genes are associated with the regulation of inflammatory response, regulation of cell–cell adhesion, positive regulation of defense response, leukocyte cell–cell adhesion and regulation of leukocyte cell–cell adhesion. While at the same time, evidenced showed that gut dysbiosis not only promoted the proliferation and migration of endometrial stromal cells in endometriotic lesions, but also promoted macrophages’ presence as well as M0 to M2 transition. This further clarify the significance of our study.

Studies have shown decreased diversity and imbalanced composition of gut flora in patients with EMs (Svensson et al., 2021). An report highlighted significant alterations in gut microbiota diversity and composition in women with endometriosis by evaluating alpha and beta diversity measures across 11 studies involving 1,727 women (Yuanyue et al., 2025). Both alpha and beta diversities were higher in controls than in patients (Svensson et al., 2021). It is worth noting that, one of the differential bacteria identified in this study, Adlercreutzia, overlapped with the protective genera we screened out. From the results of the overlapping analyses mentioned above, it can be seen that, although five key protective genera were identified in this study, whether these genera are generally absent in patients with low diversity remains to be analyzed through the big data. Whether there is a possible “keystone species” such as *Helicobacter pylori*-to gastric cancer, or a combined effect of microbial ecology of disturbed bacterial balance needs further investigaion. Altered intestinal flora diversity may result in: altered metabolite profiles, diminished immune regulation, and impaired intestinal barrier integrity, which may contribute to the occurrence and development of EMs (Sobstyl et al., 2023).

The effects of gut bacteria on host pathology and immune processes are mediated through a variety of mechanisms. Bacteria can break down indigestible nutrients into biologically active metabolites, including short-chain fatty acids (SCFAs) (Kho and Lal, 2018) SCFAs have been shown to have antiproliferative effects (Semaan et al., 2020) and anti-inflammatory properties (Parada Venegas et al., 2019). Fecal levels of short-chain fatty acids and n-butyric acid in mice with endometriosis were lower than in mice without the disease, and treatment with n-butyrate resulted in a decrease in the growth of both mouse and human endometriotic lesions (Vallvé-Juanico et al., 2019). In addition, the altered composition of the gut microbiota in endometriosis can lead to disruption of intestinal tight junctions and reduced expression of tight junction protein 2 (ZO-2) (Meroni et al., 2019), resulting in a compromised intestinal barrier. On the other hand, LPS activates macrophage TLR4 in innate immunity, which produces significant levels of TNF-alpha and IL-8 and triggers an inflammatory environment (Nothnick, 2001). TNF-alpha and IL-8 are essential for the induction of endometrial tissue adhesion and angiogenesis (Wanderley et al., 2018). In all, the impact of the diversity of microbiom is not only limited to the number and species, but also a reflection of functions diversity.

In addition, this study identified three biomarkers (AOC3, FABP4, NEK2). Among them, AOC3 and FABP4 were significantly downregulated in EMs samples, while NEK2 was significantly upregulated in EMs samples (*p* < 0.05). NEK2 (NIMA related kinase 2): NEK2 is a kinase that plays a critical role in cell cycle regulation, mainly involved in multiple important processes of human cell division, especially playing an indispensable role in mitosis (Huang et al., 2022; Gu et al., 2024; Xia et al., 2025). Although there is discrepancy between the high expression of NEK2 in bioinformatics analysis, the low expression observed in PCR analysis in our study may stem from the heterogeneity between public database samples and clinical samples in this study (such as disease subtypes, cell composition) as well as technical detection variables. Recent investigations have uncovered that NEK2 modulates the progression of EMs and associated decidualization defects via FOXO1 phosphorylation, offering a novel therapeutic target for EMs (Wang et al., 2024). This discovery aligns closely with our study’s findings, wherein NEK2 was also found to be markedly upregulated in EMs, further validating NEK2’s promising potential as a therapeutic target for this disease. AOC3 (amine oxidase, copper containing 3): AOC3 (amine oxidase, copper-containing 3), otherwise termed vascular adhesion protein 1 (VAP-1), is a molecule endowed with adhesive and enzymatic properties, as well as pro-inflammatory effects and multifunctional characteristics. It exerts a pivotal function in oxidative stress and inflammatory responses, catalyzing the oxidative deamination of amines to generate aldehydes, hydrogen peroxide, and ammonia. These products can markedly elevate the concentrations of reactive oxygen species (ROS) and lipid peroxidation products, thus aggravating the inflammatory reactions (Thézénas et al., 2020; Zhong et al., 2023; Petzinna et al., 2024). In the study of EMs, AOC3 has been reported as a novel pro-inflammatory marker of oxidative stress in peritoneal EMs lesions (Thézénas et al., 2020). Specifically, AOC3 expression is upregulated in ectopic lesions of EMs patients, which may participate in the formation and development of lesions by promoting oxidative stress and inflammatory response. However, in this study, it was found that AOC3 was significantly downregulated in EMs samples, and this difference may be related to sample heterogeneity, such as disease stage, lesion location, or individual differences among patients. Future research needs to further explore the mechanism of action of AOC3 in EMs to validate this discovery and clarify its specific role in the disease. FABP4(Fatty acid-binding protein-4): FABP4 It is a multifunctional adipokine that widely affects immune and metabolic processes (Shi Y. et al., 2023). More and more studies have shown that FABP4 dysfunction is associated with multiple metabolic syndromes, including obesity, diabetes, cardiovascular disease and metabolic inflammation (Liu et al., 2022; Su et al., 2022; Lv et al., 2023; Zhou et al., 2023b; van der Ark-Vonk et al., 2024). Clinical research revealed that adipose tissue adjacent to EMs lesions showed elevated expression levels of FABP4 and vascular endothelial growth factor (VEGF), implying that FABP4 might induce EMs through promoting adipose fibrosis (Kubo et al., 2021). However, it was found in this study that FABP4 was significantly downregulated in EMs samples, which may be related to differences in study design and methodology. Future research needs to further explore the mechanism of FABP4 in EMs to clarify its specific role in disease.

Functional compositions of sample microbial communities in previouse study predicted greatest abundance in amino acid transport and metabolism (Wei et al., 2023). Consistently, in our study, we predicted four key metabolites associated with (6-[(4R,5S)-5-methyl-2-oxoimidazolidin-4-yl]hexanoic acid, 7-Aminoheptanoic acid, N-acetylputrescine, Phenylacetic Acid), constructing a key biomarker-key metabolite-key microbe network. In the study of key metabolites, N-acetylputrescine, as a polyamine metabolite, has demonstrated significant research value. For the first time, it has been discovered that AOC3 has binding activity to the target EMs, with a binding energy of −4.9 kcal/mol. This discovery provides new clues for exploring the pathogenesis and potential diagnostic and therapeutic methods of EMs. It is worth noting that this metabolite has been proven to have diagnostic value in other diseases such as Parkinson’s disease (PD) (Peng et al., 2024), suggesting that N-acetylputrescine may also serve as a novel biomarker for EMs, providing new directions for early diagnosis and disease monitoring. The experimental data confirmed that phenylacetic acid showed high-affinity binding with AOC3, 6-[(4R,5S)-5-methyl-2-oxoimidazolidin-4-yl]hexanoic acid and NEK2, and all of them conformed to the Lipinski-like pharmacophore rule, suggesting that the above metabolites can be used as candidate lead compounds of EMs, which can provide preliminary drug candidates for future mechanistic and pharmacological validation. In the future, based on the metabolite-target binding mode discovered in this study, structure optimization and new drug creation can be carried out to develop specific targeted drugs against AOC3, FABP4, and NEK2, which can provide a new drug intervention strategy for EMs.6-[(4R,5S)-5-methyl-2-oxoimidazolidin-4-yl] hexanoic acid.

Especially phenylacetic acid may inhibit the activity of AOC3 enzyme to reduce the release of inflammatory factors (Boyer et al., 2021), and may also bind to FABP4 and inhibit its function to block downstream inflammatory signaling (Mao et al., 2021). At the same time, it may downregulate the expression or activity of NEK2 to inhibit ERK/MAPK signaling (Xing et al., 2021), thereby reducing the inflammatory state of EMs lesions. These findings not only offer a novel perspective for investigating the molecular mechanisms of EMs, but also hold the potential to uncover new targets for diagnosing and treating this disease. Future research will delve deeper into the specific mode of action of NAP in EMs and its clinical applicability during disease progression.

Our study invited the innovative integration of multidisciplinary methods: For the first time, Mendelian randomization and network pharmacology were systematically combined for the study of the relationship between metabolites of gut microbiome and endometriosis. The combination of Mendelian randomization (MR) and network pharmacology (NP) provides evidence of causality and overcomes the limitations of traditional observational studies of confounding factors and reverse causation, while network pharmacology reveals the underlying mechanisms of action at the systemic level. This combination realizes a seamless transition from “causal inference” to “mechanism exploration,” and the research logic is very rigorous. Secondly, instead of merely identifying the bacterial groups that are causally related to EMs, a complete network of pathways has been constructed. This links the microbiome, metabolome, and host gene expression at three levels, providing a new, systematic perspective for understanding the pathophysiology of EMs that goes beyond previous studies focusing on a single level. At last, a combination of public database mining, bioinformatics prediction, and own RT-qPCR clinical sample validation. This multilevel research strategy enhanced the reliability of the findings. Although the expression trend of NEK2 was inconsistent in the validation, honestly reporting and discussing such discrepancies instead demonstrated the rigor and scientific validity of the study and pointed the way for future research.

This study successfully identified three EMs biomarkers associated with GM metabolites (AOC3, FABP4, NEK2). Subsequently, we predicted four key metabolites associated with biomarkers (6-[(4R, 5S)-5-methyl-2-oxoimidazolidin-4-yl] hexanoic acid, 7-Aminoheptanoic acid, N-acetylpurine, Phenylacetic Acid) and constructed a network of biomarkers key metabolites key microorganisms. Simultaneously, druglike evaluations and molecular docking analyses were performed on these key metabolites, striving to offer a novel perspective for the treatment of EMs. However, this study still harbors certain limitations: 1. The GWAS data were mainly from people of European ancestry, which improved the reliability of causal inference in the context of the same race, but limited the universality of the results. In addition, MR design cannot completely exclude population stratification, and the sample size and statistical efficacy are insufficient, which may not fully reveal the potential causal relationship. 2. The expression of AOC3, FABP4 and NEK2 in clinical samples was only verified by RT-qPCR, and no interventional experiments were carried out to elucidate their specific mechanism of action in endometriosis. 3. The identified gut microbiota and metabolites have not been directly experimentally validated. Therefore, the proposed gut microbiota-metabolite-endometriosis axis is mainly based on computer prediction, and still needs to be further verified by microbial sequencing, targeted metabolomics, and functional studies. This study is essentially a computational and hypothetical generative study, and there is a lack of direct evidence for *in vitro* cell experiments, *in vivo* animal models, and clinical intervention trials for screening metabolites. The significance of this study is that the screened AOC3, FABP4 and NEK2 can be used as biomarkers for precise classification and personalized treatment of EMs for early clinical screening, disease grading and prognosis assessment. Clinically, individualized plans can be formulated based on the characteristics of the patient’s intestinal flora, metabolite levels and marker expression profiles, and the microbiota regulation can be implemented for patients with dysbiosis, and targeted drugs can be used for patients with abnormal targets to make up for the shortcomings of existing treatment. In addition, protective flora such as *Bifidobacterium bifidum* and Senegalimassilia identified in this study may serve as candidate microbial targets for future research on probiotics or microbiota regulation, but their therapeutic potential needs to be verified in prospective clinical cohorts and experimental models.

In the future, we can restore the diversity of intestinal flora in patients by means of probiotic supplementation, prebiotic modulation and fecal transplantation, up-regulate the levels of key metabolites such as N-acetylputrescine and phenylacetic acid, and regulate the expression of biomarkers to ameliorate the inflammation and formation of ectopic foci, so as to promote the clinical translation of the colony-targeted therapies. Overall, we will continue to delve into the relevant research on EMs and further optimize and validate our findings.

## Data Availability

All research data and Supplementary materials for this study have been deposited in FigShare and are publicly available at doi: https://doi.org/10.6084/m9.figshare.32520402.

## Footnotes

1
https://gwas.mrcieu.ac.uk/

2
https://www.mibiogen.org

3
https://www.ncbi.nlm.nih.gov/geo/

4
https://www.disgenet.org/

5
https://www.genecards.org/

6
https://string-db.org/

7
http://genemania.org/

8
http://bio-bigdata.cn/mRNALocater/

9
http://www.swissadme.ch/

10
https://admetlab3.scbdd.com/server/screening

11
http://www.rcsb.org

12
https://cadd.labshare.cn/cb-dock/php/blinddock.php
