# Supplementary material for: Exploring the potential of gut microbiota metabolites in the treatment of endometriosis through network pharmacology and Mendelian randomization
Source: Front Microbiol. 2026 Jun 11;17:1733323. doi: 10.3389/fmicb.2026.1733323 (PMC13294049; doi:10.3389/fmicb.2026.1733323)
Supplement: Supplementary file 1 [file Data_Sheet_1.ZIP › Supplementary figures/Supplementary figures/S1/genus.Allisonella.id.2174.pdf]

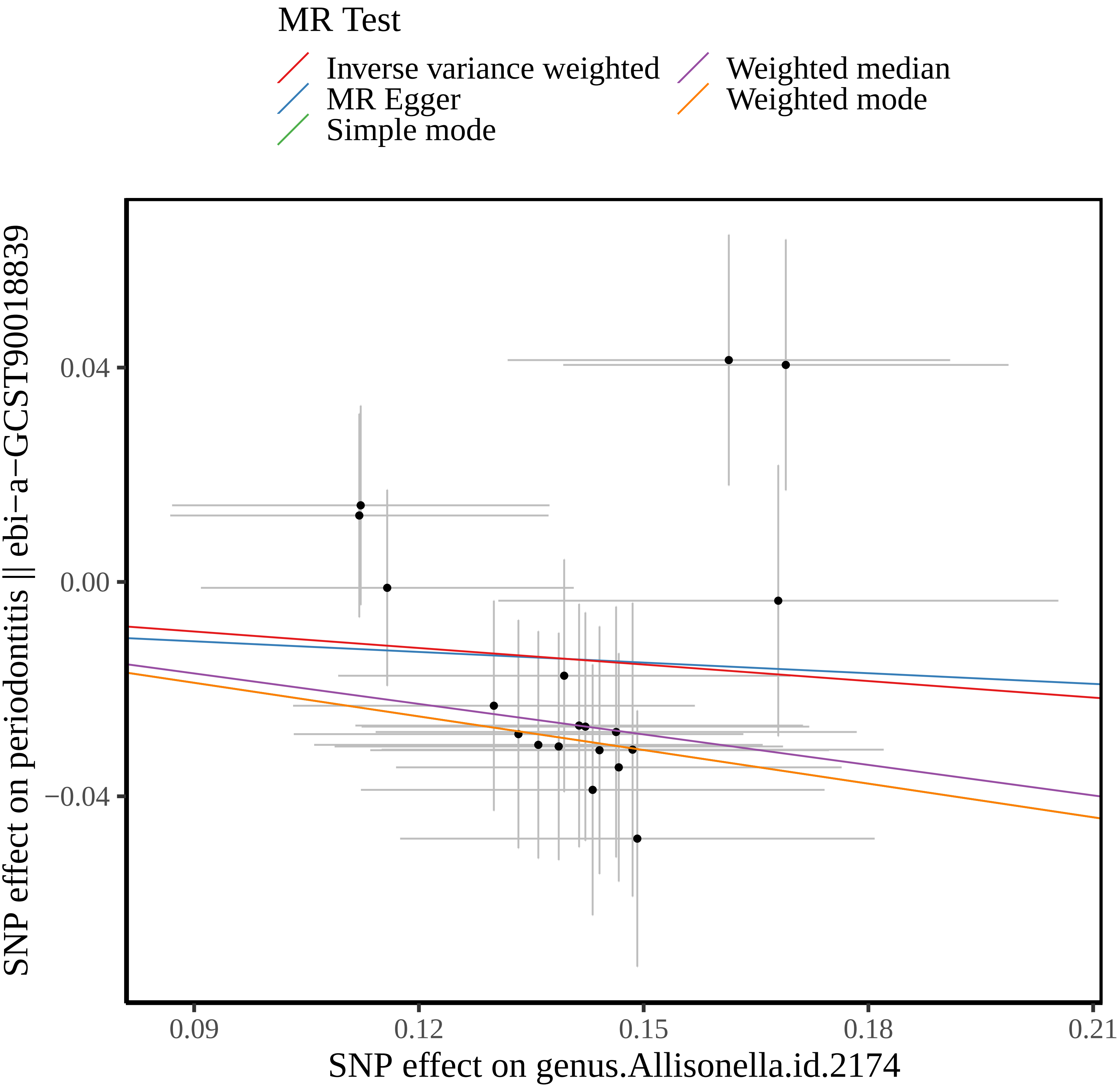
MR Test
Inverse variance weighted
MR Egger
Simple mode
Weighted median
Weighted mode
SNP effect on periodontitis || ebi−a−GCST90018839
0.04
0.00
−0.04
0.09
0.12
0.15
0.18
0.21
SNP effect on genus.Allisonella.id.2174
