# Supplementary material for: Exploring the potential of gut microbiota metabolites in the treatment of endometriosis through network pharmacology and Mendelian randomization
Source: Front Microbiol. 2026 Jun 11;17:1733323. doi: 10.3389/fmicb.2026.1733323 (PMC13294049; doi:10.3389/fmicb.2026.1733323)
Supplement: Supplementary file 1 [file Data_Sheet_1.ZIP › Supplementary figures/Supplementary figures/S1/genus.RuminococcaceaeUCG004.id.11362.pdf]

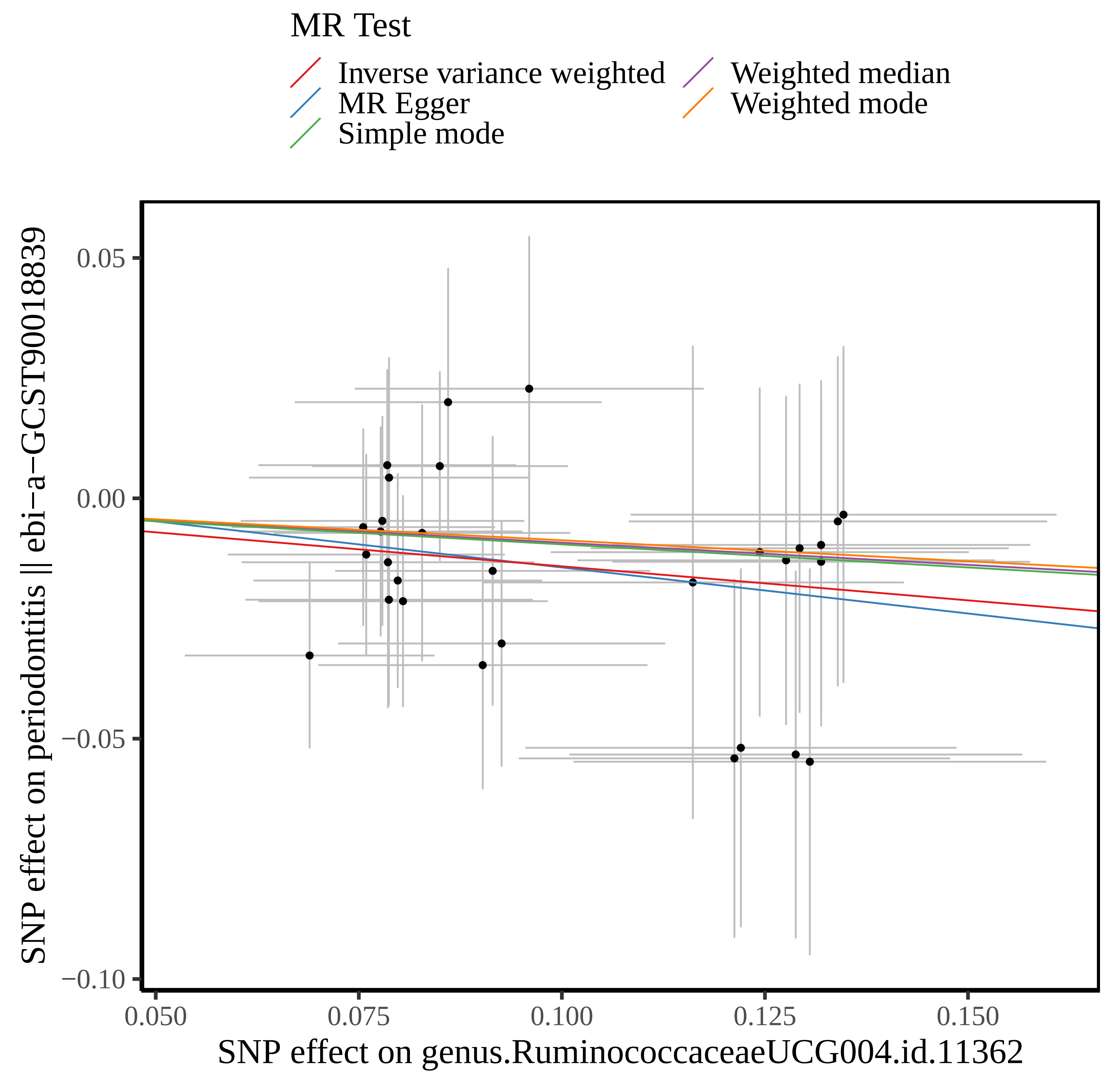
MR Test
Inverse variance weighted
MR Egger
Simple mode
Weighted median
Weighted mode
0.05
0.00
−0.05
−0.10
0.050
0.075
0.100
0.125
0.150
SNP effect on periodontitis || ebi−a−GCST90018839
SNP effect on genus.RuminococcaceaeUCG004.id.11362
