# Supplementary material for: Exploring the potential of gut microbiota metabolites in the treatment of endometriosis through network pharmacology and Mendelian randomization
Source: Front Microbiol. 2026 Jun 11;17:1733323. doi: 10.3389/fmicb.2026.1733323 (PMC13294049; doi:10.3389/fmicb.2026.1733323)
Supplement: Supplementary file 1 [file Data_Sheet_1.ZIP › Supplementary figures/Supplementary figures/S2/genus.RuminococcaceaeUCG004.id.11362.pdf]

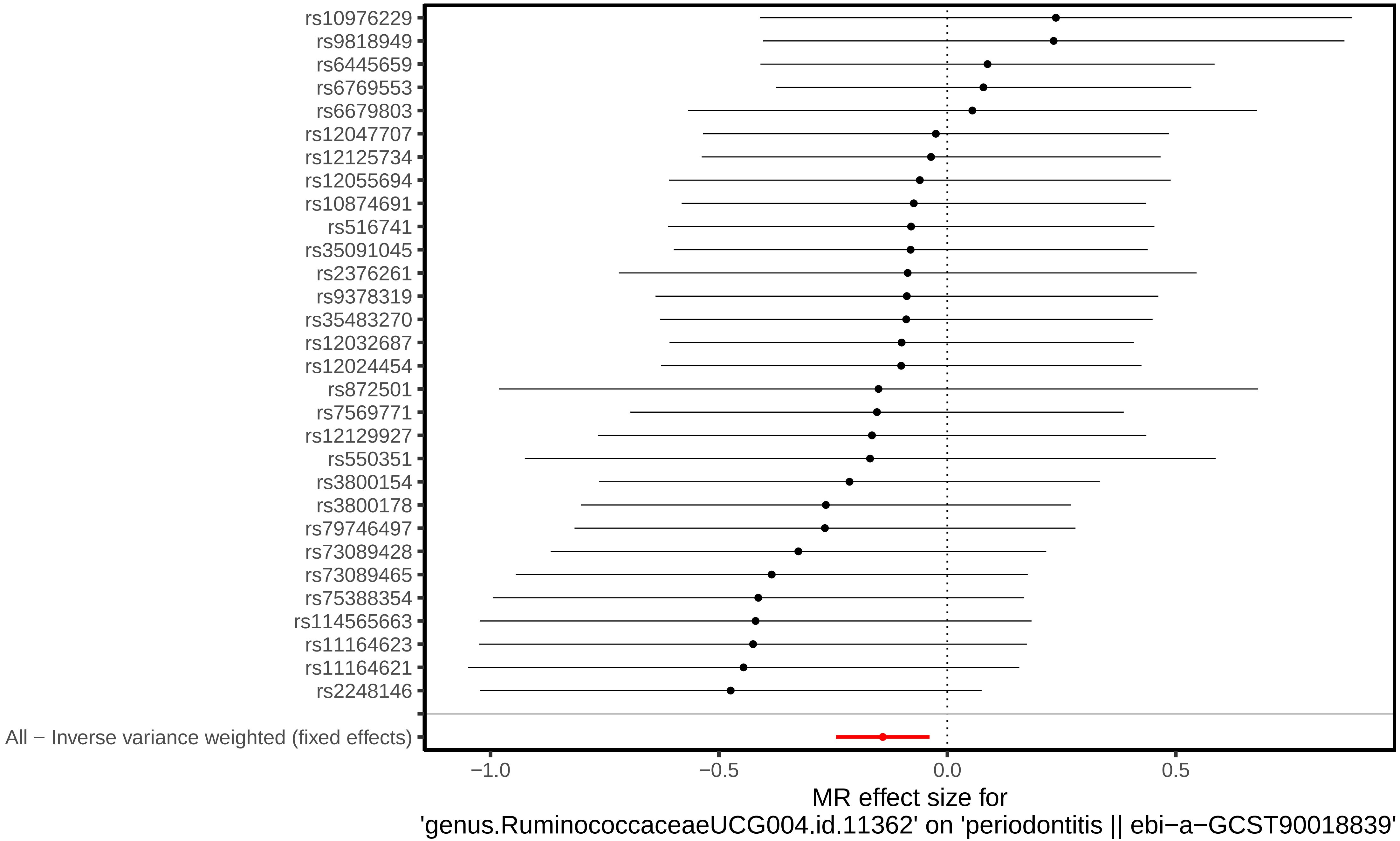

rs10976229
rs9818949
rs6445659
rs6769553
rs6679803
rs12047707
rs12125734
rs12055694
rs10874691
rs516741
rs35091045
rs2376261
rs9378319
rs35483270
rs12032687
rs12024454
rs872501
rs7569771
rs12129927
rs550351
rs3800154
rs3800178
rs79746497
rs73089428
rs73089465
rs75388354
rs114565663
rs11164623
rs11164621
rs2248146
All − Inverse variance weighted (fixed effects)
−1.0
−0.5
0.0
0.5
MR effect size for
'genus.RuminococcaceaeUCG004.id.11362' on 'periodontitis || ebi−a−GCST90018839'
