# Supplementary material for: Exploring the potential of gut microbiota metabolites in the treatment of endometriosis through network pharmacology and Mendelian randomization
Source: Front Microbiol. 2026 Jun 11;17:1733323. doi: 10.3389/fmicb.2026.1733323 (PMC13294049; doi:10.3389/fmicb.2026.1733323)
Supplement: Supplementary file 1 [file Data_Sheet_1.ZIP › Supplementary figures/Supplementary figures/S4/genus.Intestinibacter.id.11345.pdf]

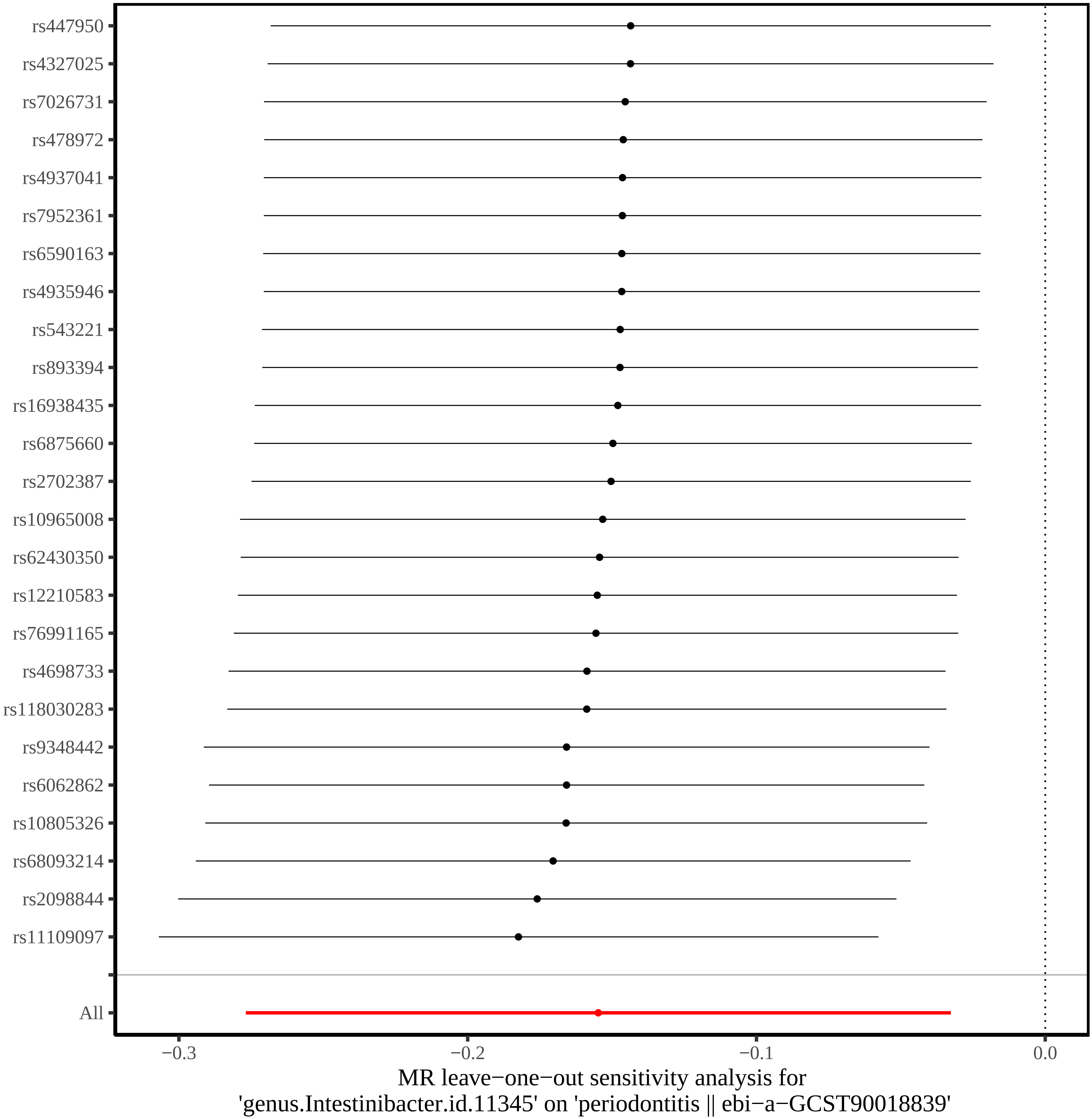

rs447950
rs4327025
rs7026731
rs478972
rs4937041
rs7952361
rs6590163
rs4935946
rs543221
rs893394
rs16938435
rs6875660
rs2702387
rs10965008
rs62430350
rs12210583
rs76991165
rs4698733
rs118030283
rs9348442
rs6062862
rs10805326
rs68093214
rs2098844
rs11109097
All
−0.3
−0.2
−0.1
0.0
MR leave−one−out sensitivity analysis for
'genus.Intestinibacter.id.11345' on 'periodontitis || ebi−a−GCST90018839'
