# Supplementary figures and images for: Exploring the potential of gut microbiota metabolites in the treatment of endometriosis through network pharmacology and Mendelian randomization
Source: Front Microbiol. 2026 Jun 11;17:1733323. doi: 10.3389/fmicb.2026.1733323 (PMC13294049; doi:10.3389/fmicb.2026.1733323)

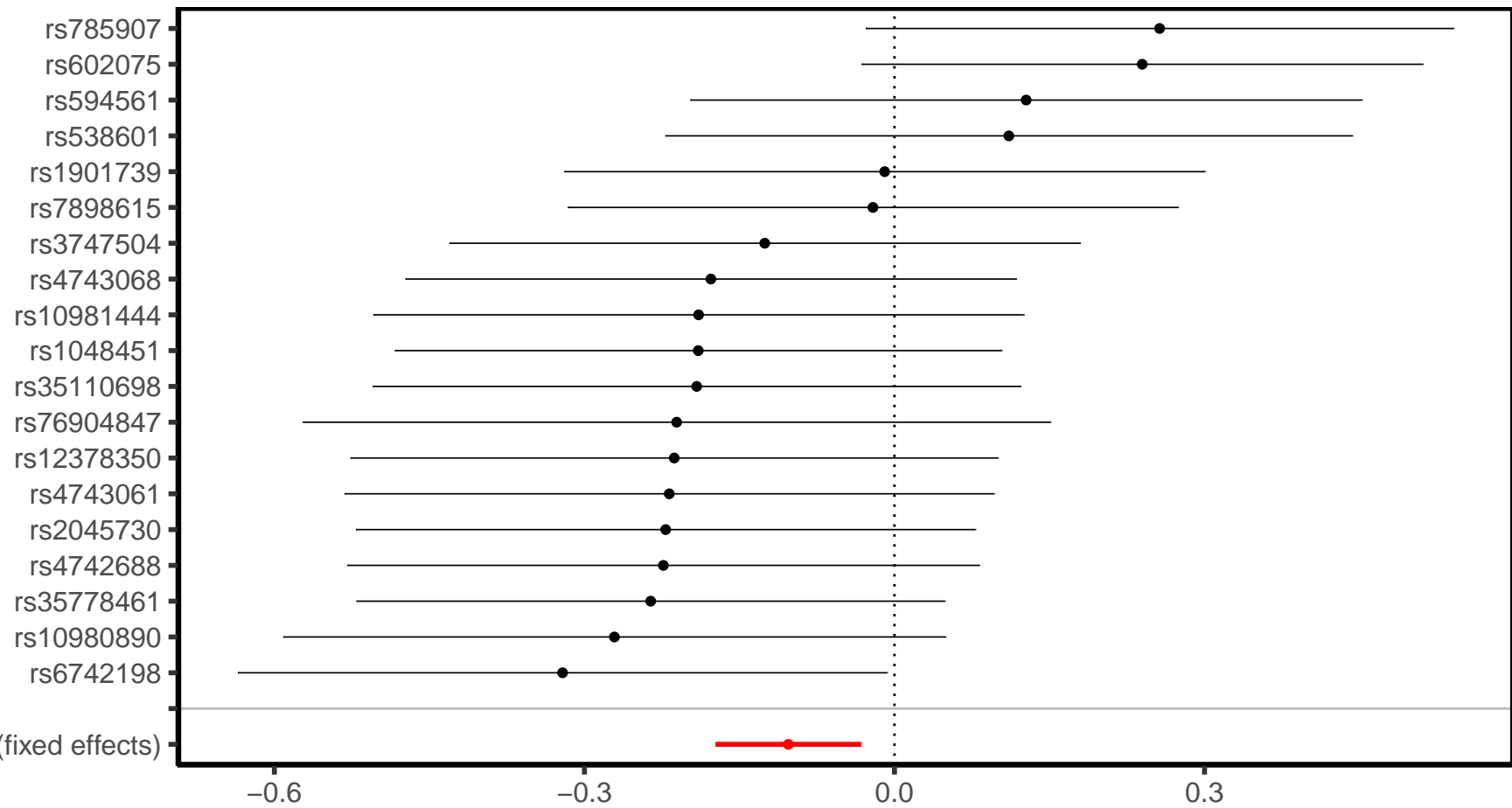

MR effect size for  
'genus.Allisonella.id.2174' on 'periodontitis || ebi-a-GCST90018839'

Supplement: Supplementary file 1 [file Data_Sheet_1.ZIP › Supplementary figures/Supplementary figures/S2/genus.Allisonella.id.2174.pdf]

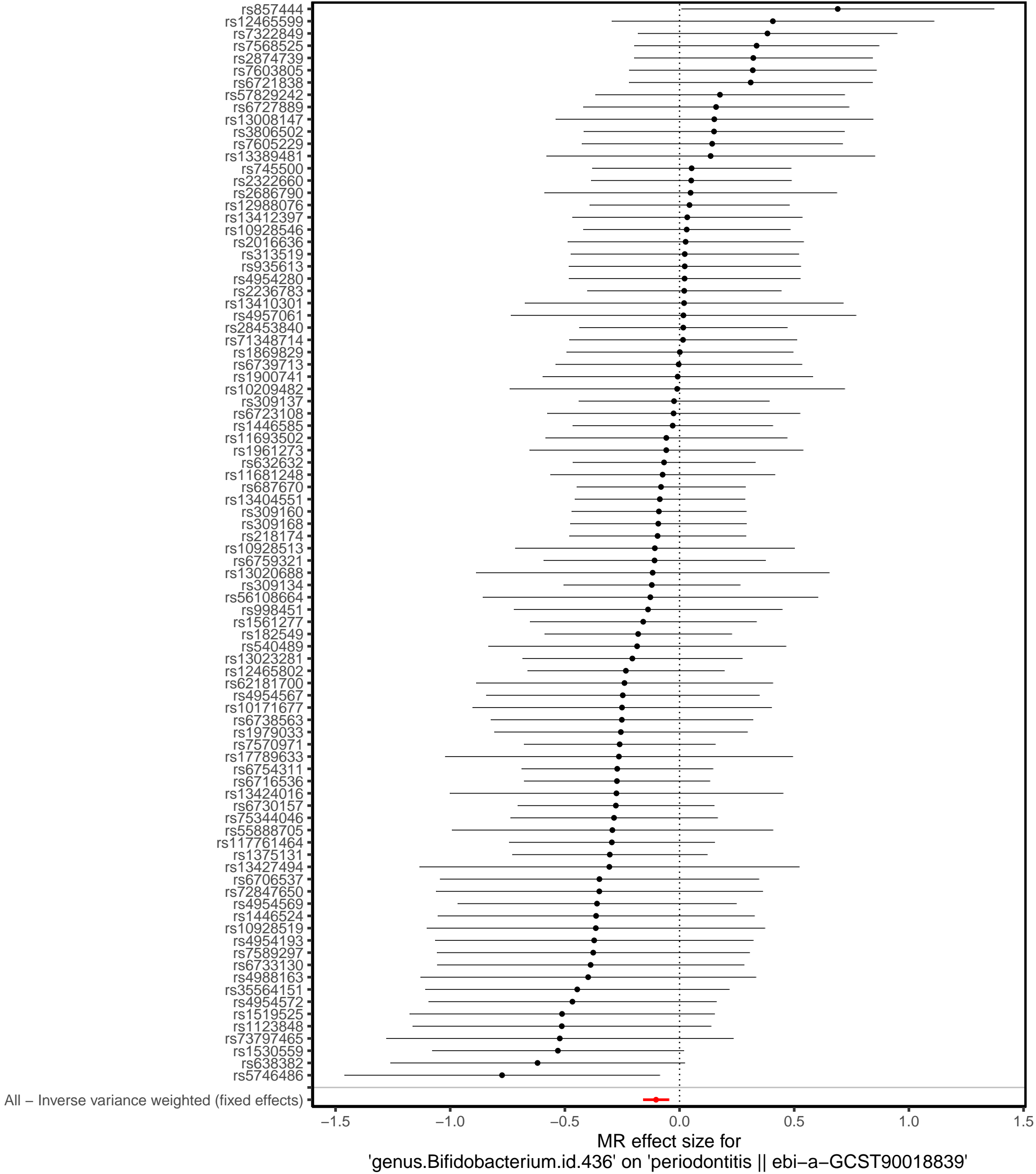

Supplement: Supplementary file 1 [file Data_Sheet_1.ZIP › Supplementary figures/Supplementary figures/S2/genus.Bifidobacterium.id.436.pdf]

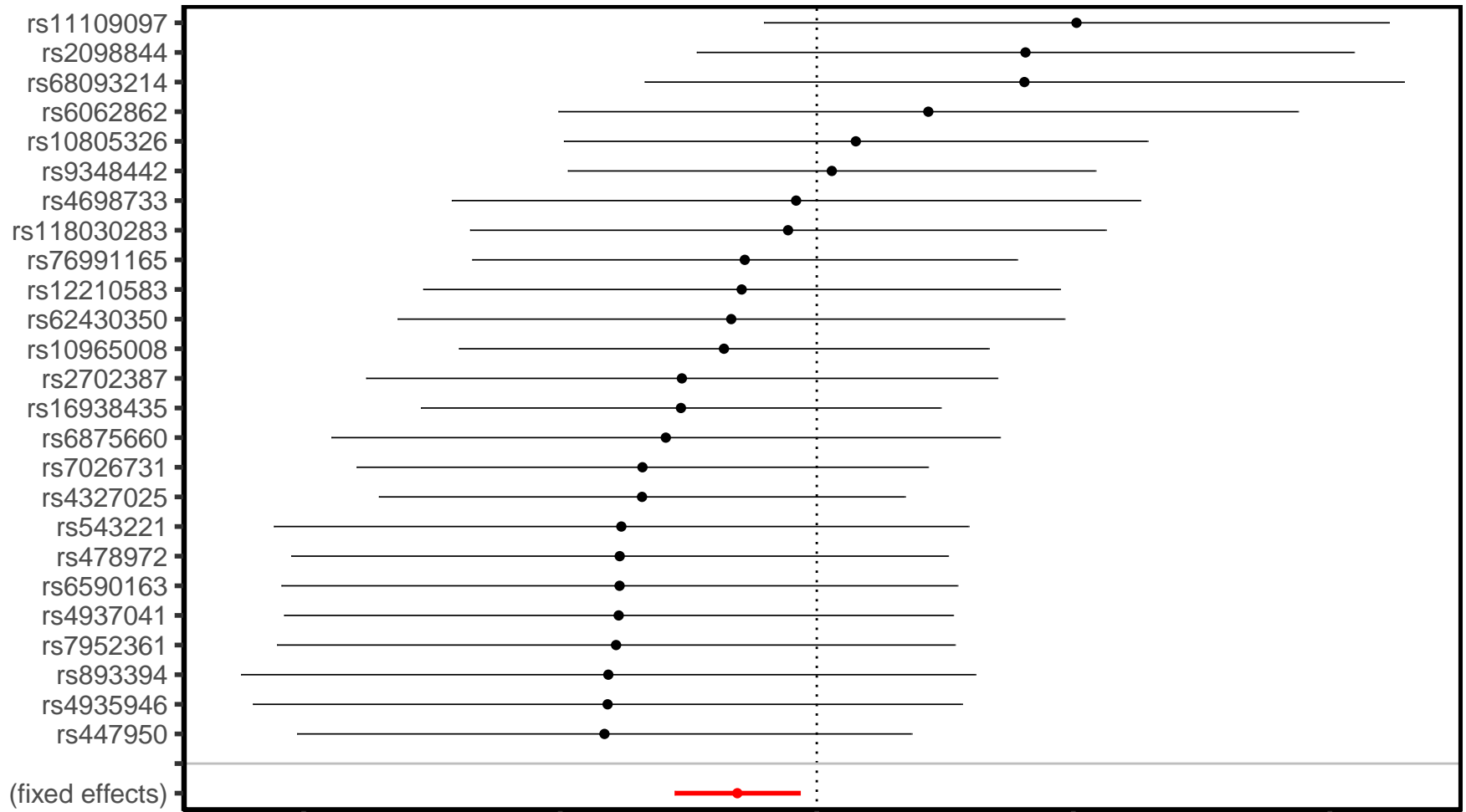

MR effect size for  
'genus.Intestinibacter.id.11345' on 'periodontitis || ebi-a-GCST90018839'

Supplement: Supplementary file 1 [file Data_Sheet_1.ZIP › Supplementary figures/Supplementary figures/S2/genus.Intestinibacter.id.11345.pdf]

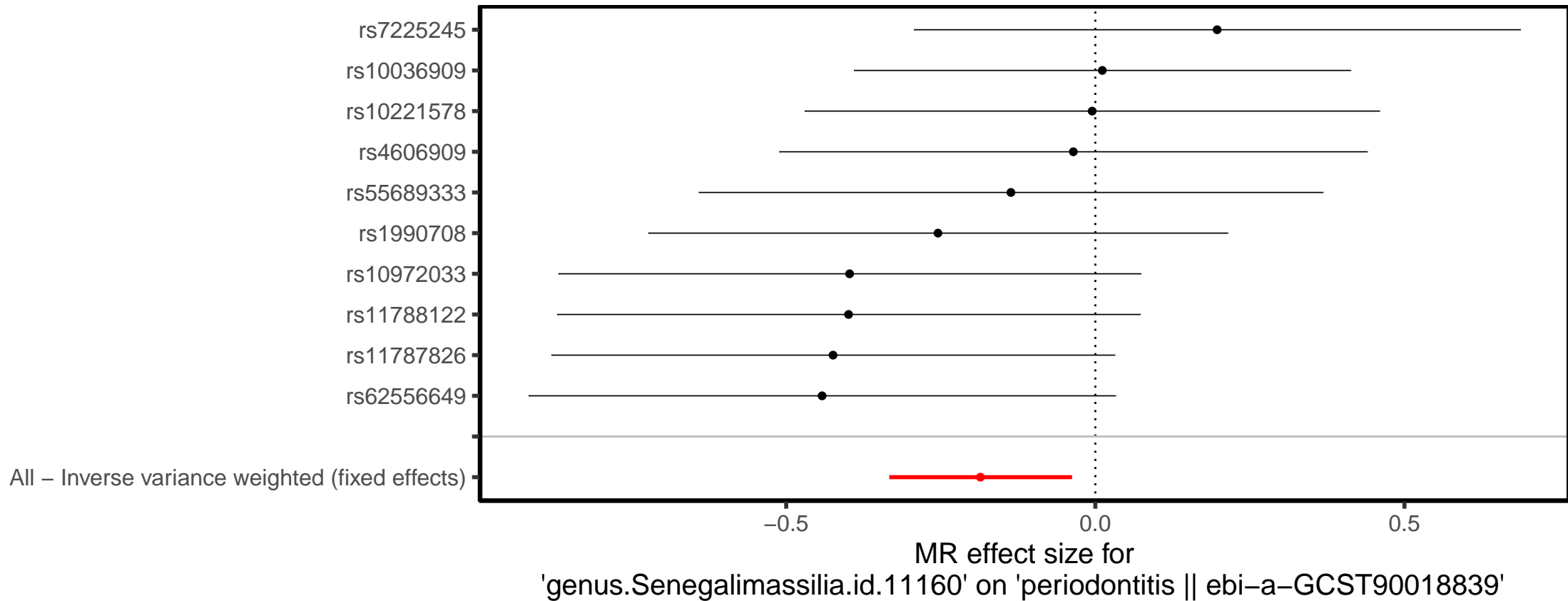

Supplement: Supplementary file 1 [file Data_Sheet_1.ZIP › Supplementary figures/Supplementary figures/S2/genus.Senegalimassilia.id.11160.pdf]

# MR Method

| Inverse variance weighted

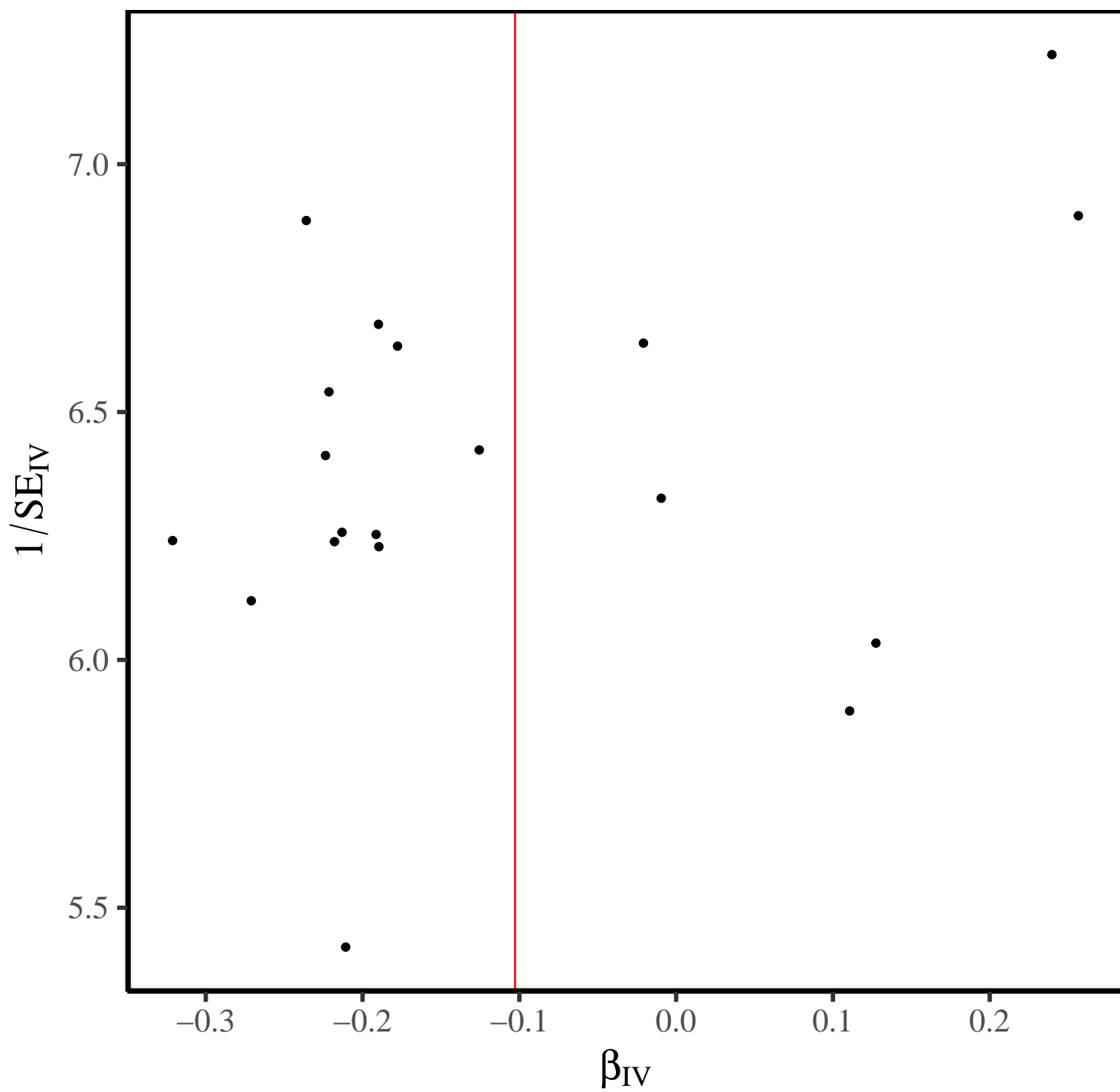

Supplement: Supplementary file 1 [file Data_Sheet_1.ZIP › Supplementary figures/Supplementary figures/S3/genus.Allisonella.id.2174.pdf]

# MR Method

| Inverse variance weighted

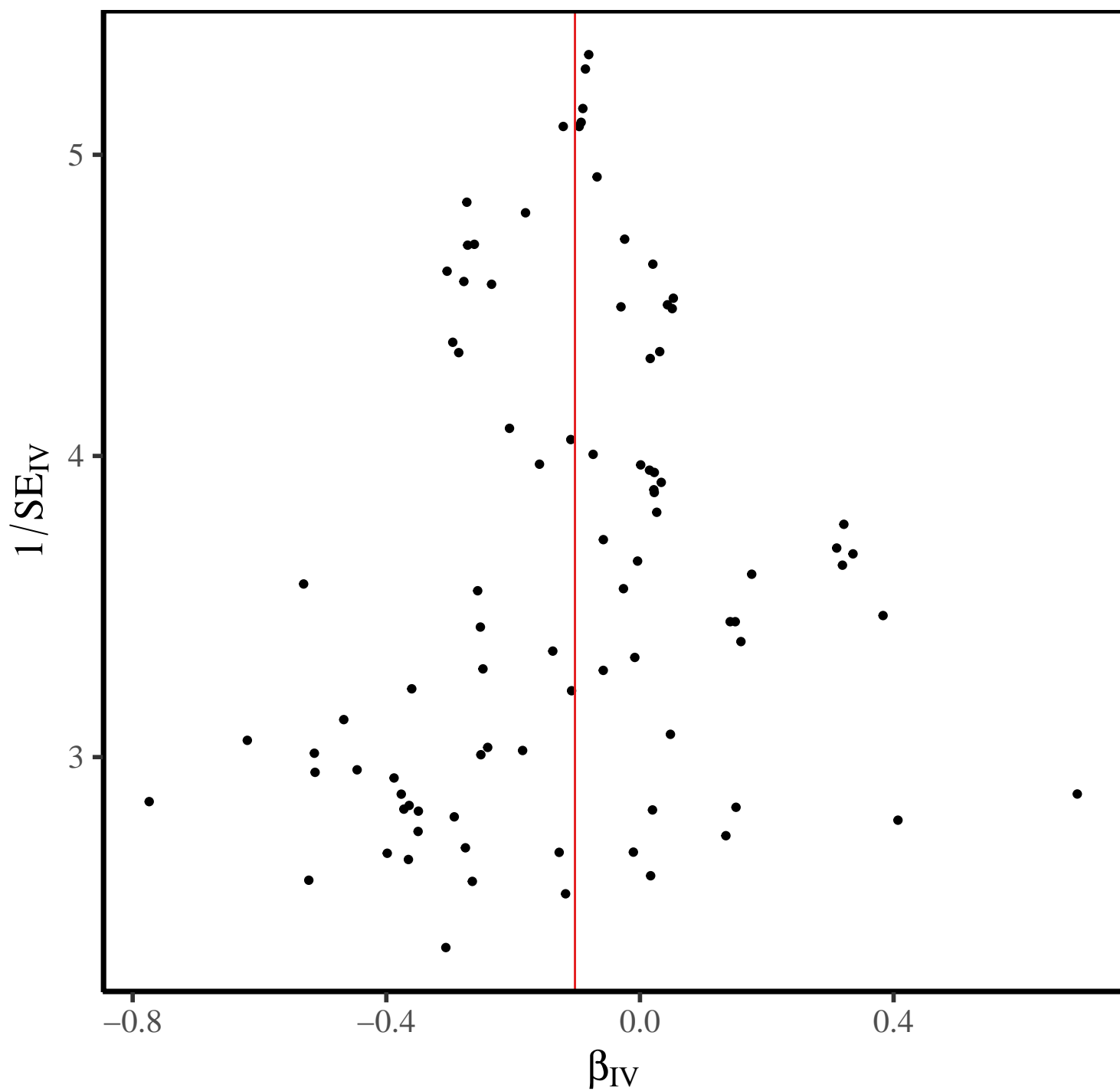

Supplement: Supplementary file 1 [file Data_Sheet_1.ZIP › Supplementary figures/Supplementary figures/S3/genus.Bifidobacterium.id.436.pdf]

# MR Method

| Inverse variance weighted

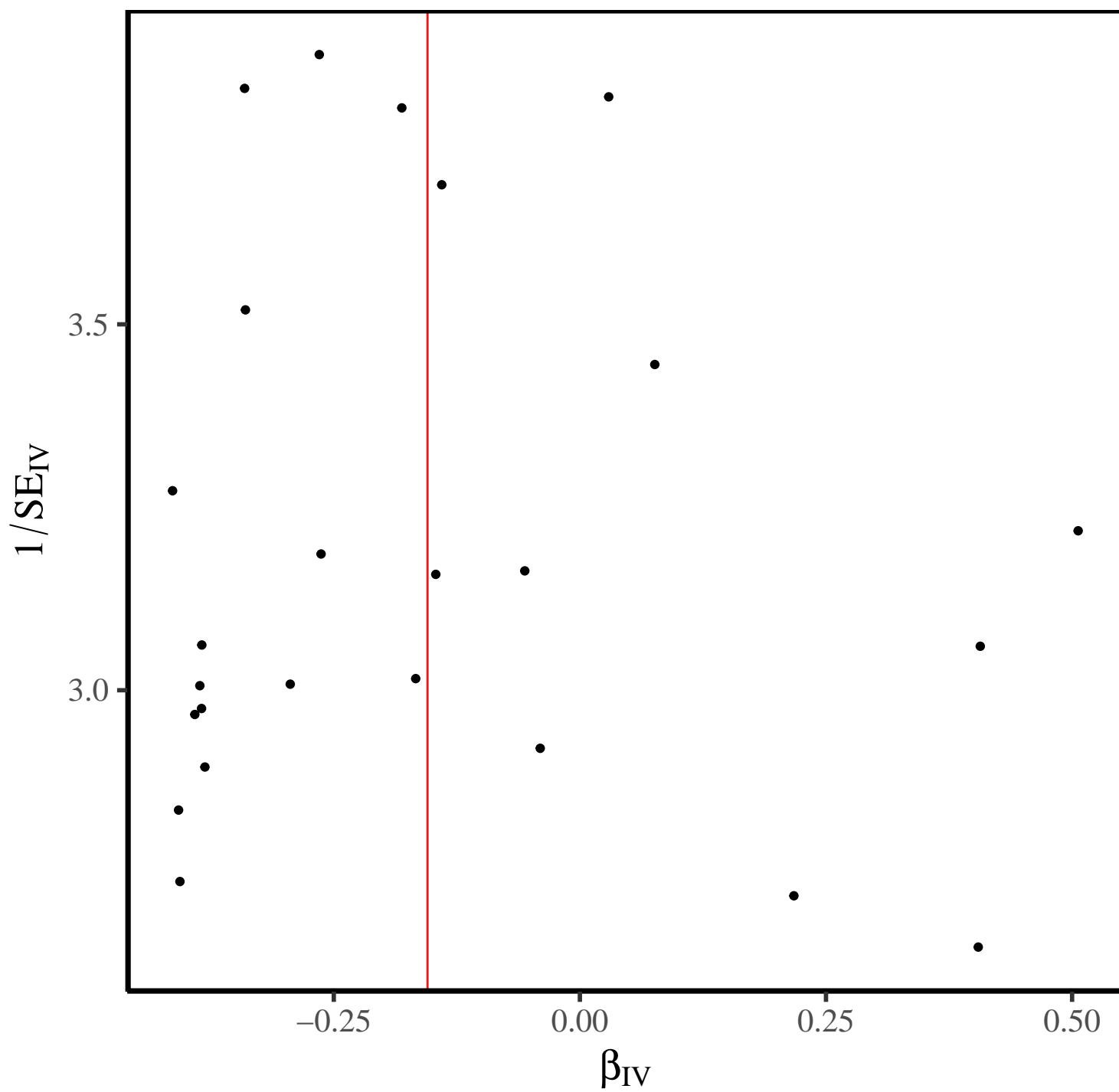

Supplement: Supplementary file 1 [file Data_Sheet_1.ZIP › Supplementary figures/Supplementary figures/S3/genus.Intestinibacter.id.11345.pdf]

# MR Method

| Inverse variance weighted

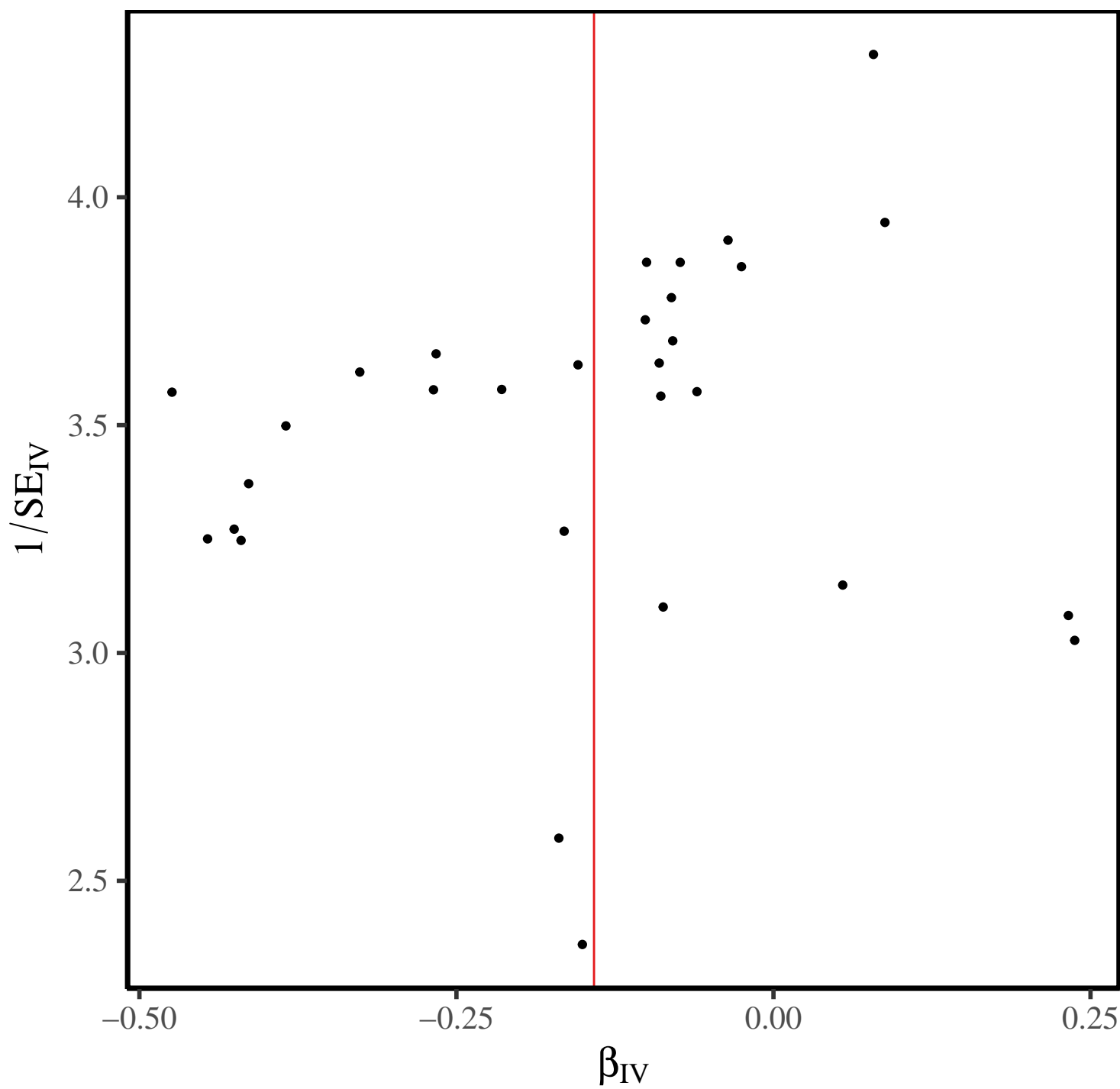

Supplement: Supplementary file 1 [file Data_Sheet_1.ZIP › Supplementary figures/Supplementary figures/S3/genus.RuminococcaceaeUCG004.id.11362.pdf]

# MR Method

| Inverse variance weighted

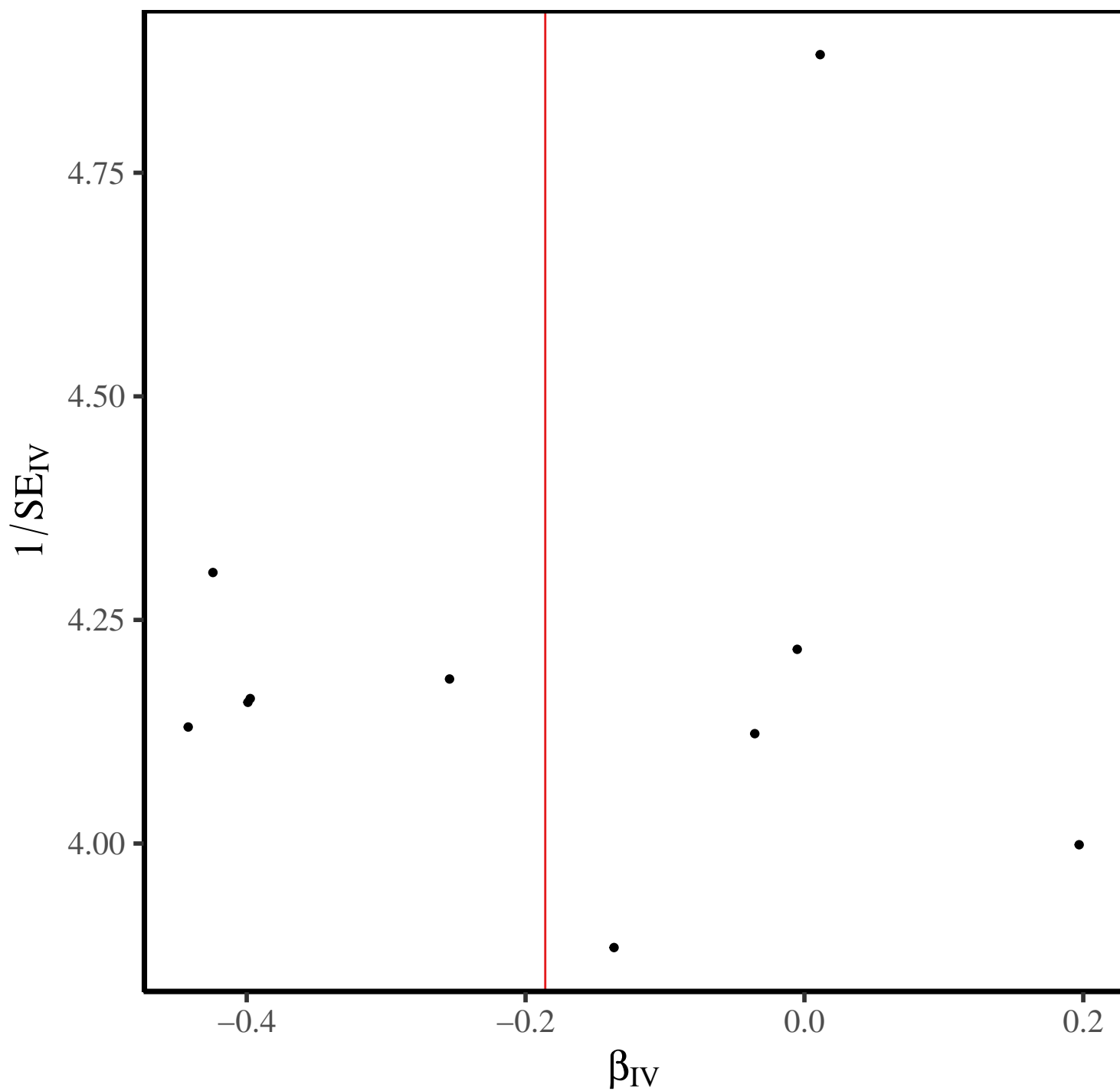

Supplement: Supplementary file 1 [file Data_Sheet_1.ZIP › Supplementary figures/Supplementary figures/S3/genus.Senegalimassilia.id.11160.pdf]

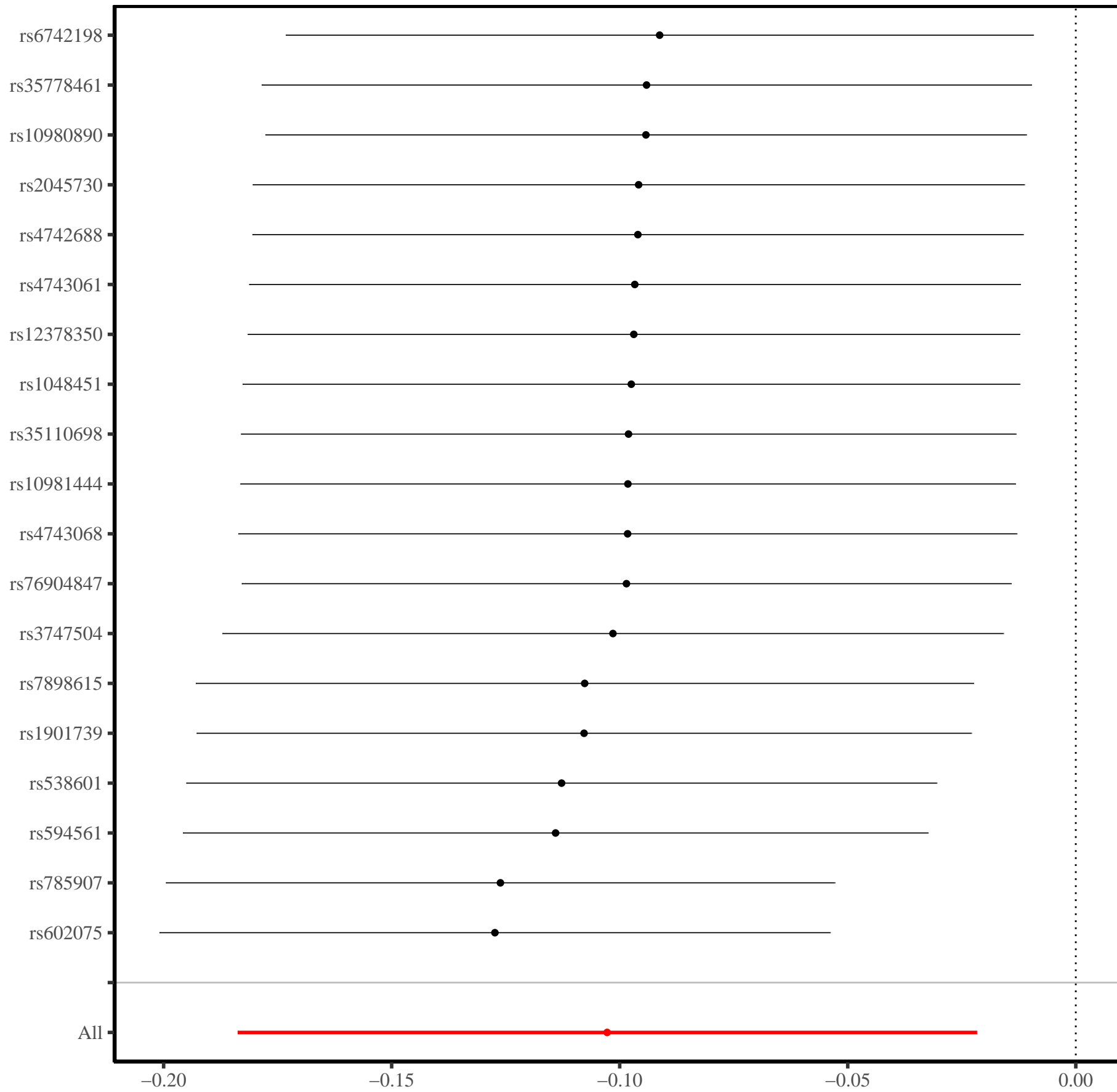

Supplement: Supplementary file 1 [file Data_Sheet_1.ZIP › Supplementary figures/Supplementary figures/S4/genus.Allisonella.id.2174.pdf]

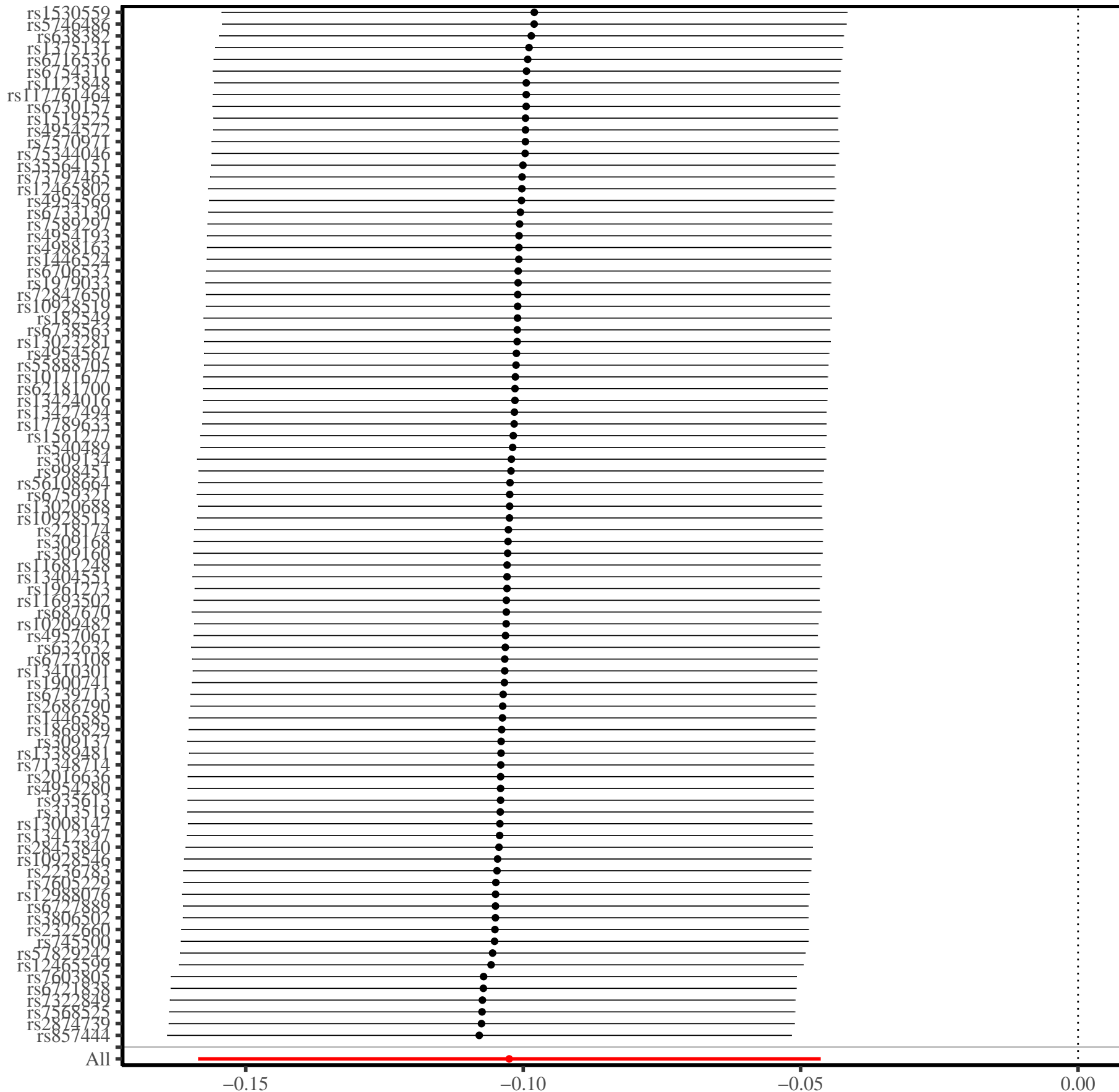

Supplement: Supplementary file 1 [file Data_Sheet_1.ZIP › Supplementary figures/Supplementary figures/S4/genus.Bifidobacterium.id.436.pdf]

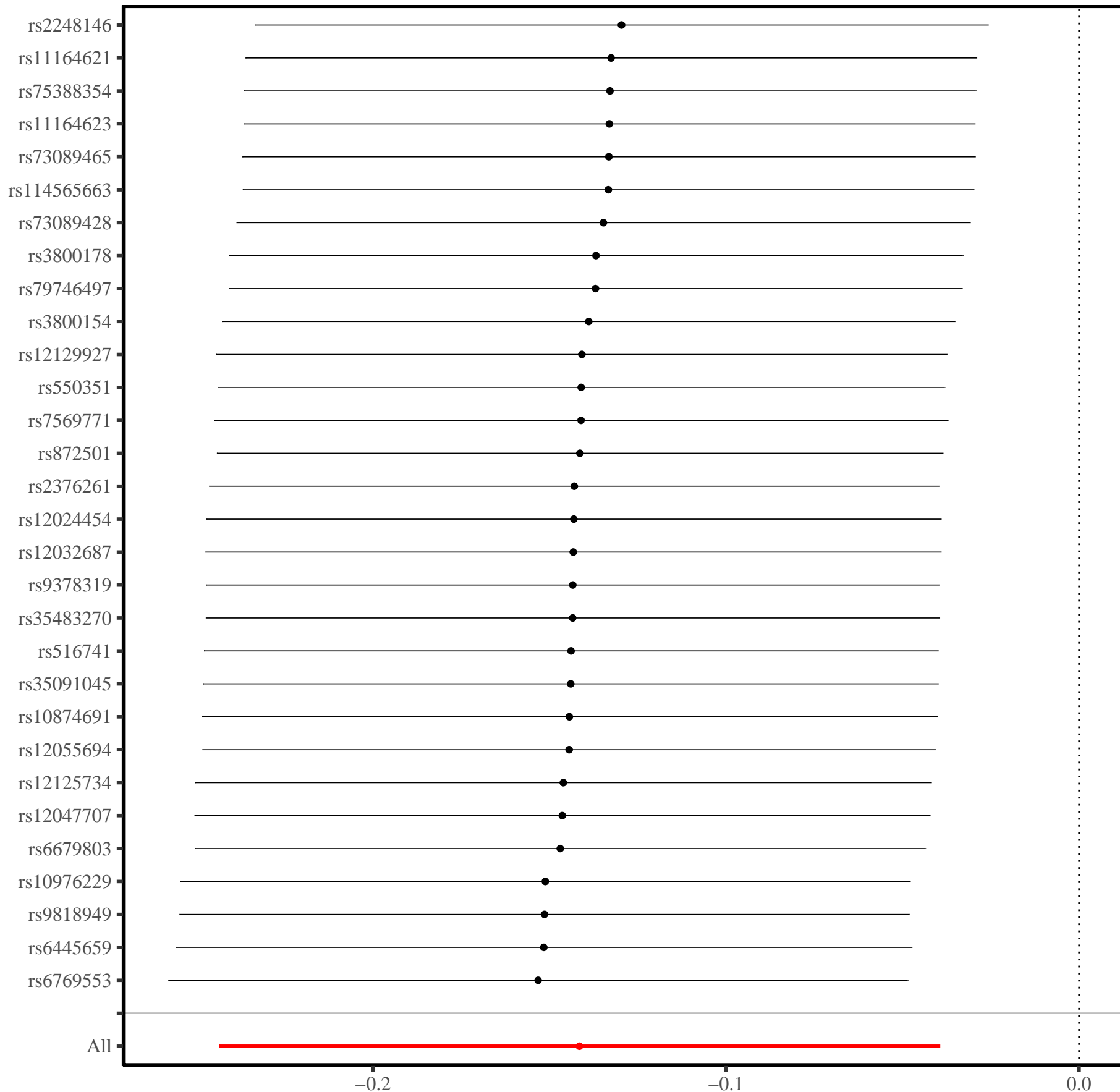

Supplement: Supplementary file 1 [file Data_Sheet_1.ZIP › Supplementary figures/Supplementary figures/S4/genus.RuminococcaceaeUCG004.id.11362.pdf]

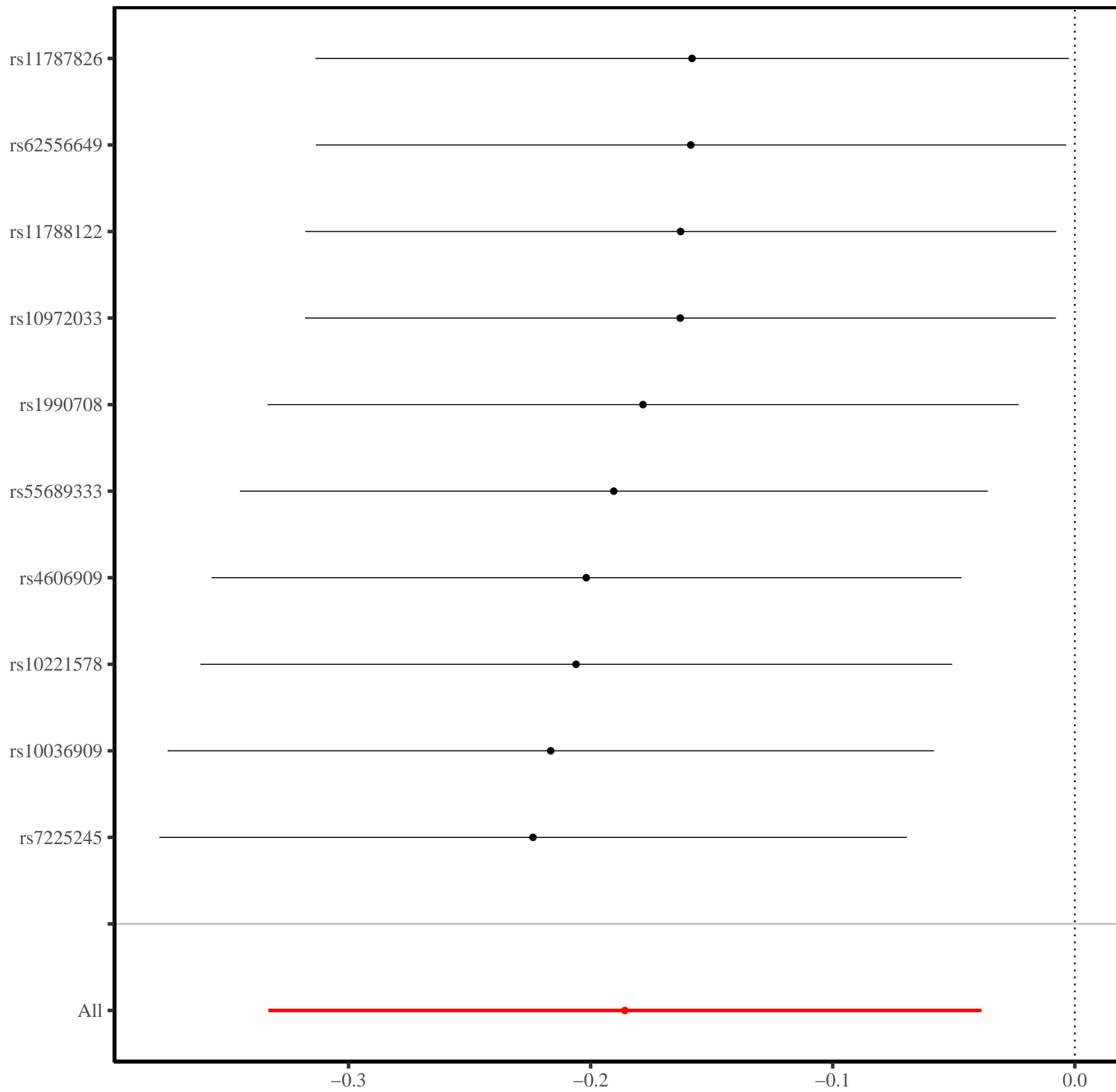

Supplement: Supplementary file 1 [file Data_Sheet_1.ZIP › Supplementary figures/Supplementary figures/S4/genus.Senegalimassilia.id.11160.pdf]
